# Air-Stable Binary Hydrated Eutectic Electrolytes with Unique Solvation Structure for Rechargeable Aluminum-Ion Batteries

**DOI:** 10.1007/s40820-023-01160-z

**Published:** 2023-07-29

**Authors:** Pengyu Meng, Jian Huang, Zhaohui Yang, Min Jiang, Yibo Wang, Wei Zhang, Jiao Zhang, Baode Sun, Chaopeng Fu

**Affiliations:** 1 School of Materials Science and Engineering, Shanghai Jiao Tong University, Shanghai, 200240 People’s Republic of China; 2 State Key Laboratory of High Performance Ceramics and Superfine Microstructures, Shanghai Institute of Ceramics, Chinese Academy of Sciences, Shanghai, 200050 People’s Republic of China; 3 Advanced Technology Institute, University of Surrey, Guildford, GU2 7XH Surrey UK

**Keywords:** Al-ion battery, Hydrated eutectic electrolyte, Mechanism, Solvation structure

## Abstract

Aluminum-ion batteries (AIBs) have been highlighted as a potential alternative to lithium-ion batteries for large-scale energy storage due to the abundant reserve, light weight, low cost, and good safety of Al. However, the development of AIBs faces challenges due to the usage of AlCl_3_-based ionic liquid electrolytes, which are expensive, corrosive, and sensitive to humidity. Here, we develop a low-cost, non-corrosive, and air-stable hydrated eutectic electrolyte composed of aluminum perchlorate nonahydrate and methylurea (MU) ligand. Through optimizing the molar ratio to achieve the unique solvation structure, the formed Al(ClO_4_)_3_·9H_2_O/MU hydrated deep eutectic electrolyte (AMHEE) with an average coordination number of 2.4 can facilely realize stable and reversible deposition/stripping of Al. When combining with vanadium oxide nanorods positive electrode, the Al-ion full battery delivers a high discharge capacity of 320 mAh g^−1^ with good capacity retention. The unique solvation structure with a low desolvation energy of the AMHEE enables Al^3+^ insertion/extraction during charge/discharge processes, which is evidenced by in situ synchrotron radiation X-ray diffraction. This work opens a new pathway of developing low-cost, safe, environmentally friendly and high-performance electrolytes for practical and sustainable AIBs.

**Supplementary Information:**

The online version contains supplementary material available at 10.1007/s40820-023-01160-z.

## Highlights

- A non-corrosive and air-stable hydrated eutectic electrolyte is developed.
- The electrolyte is composed of aluminum perchlorate nonahydrate and methylurea.
- The unique solvation structure enables reversible deposition/stripping of Al.
- The Al-ion battery in this electrolyte shows good charge/discharge performance.

**Supplementary Information:**

The online version contains supplementary material available at 10.1007/s40820-023-01160-z.

## Graphical abstract

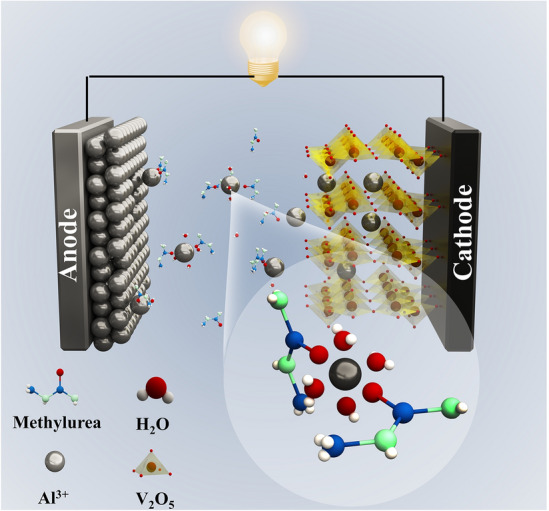

## Introduction

Rechargeable batteries for large-scale energy storage are essential to efficiently utilize renewable solar and wind energy and support peak shaving and valley filling of grid. Although Li-ion cells continue to dominate battery market, the high cost caused by the limited reserve of lithium and safety risk caused by flammable organic electrolytes retard their wide application in grid-scale energy storage [1, 2]. Al-ion batteries (AIBs) are considered as a promising candidate for large-scale electrochemical energy storage with merits of low cost, rich abundance, inherent safety and the highest volumetric capacity of Al (8.04 vs 2.06 Ah cm^−3^ of Li) [3–7]. However, the implementation of this technology still needs to overcome several technical obstacles in terms of electrolyte. Similar electrolytes based on organic solvents commonly used in LIBs are unsuitable for AIBs due to the low solubility of Al salts in organic solvents caused by the large ionic surface charge density of Al^3+^ [3]. To date, most of the AIBs are based on AlCl_3_/1-ethyl-3-methylimidazolium chloride ([EMIm]Cl) ionic liquid (IL) electrolyte, which can realize reversible plating/stripping of Al at room temperature [8–11]. However, the high cost and humidity sensitivity of AlCl_3_/[EMIm]Cl IL severely restrict the development of AIBs. Moreover, the AlCl_3_/[EMIm]Cl electrolyte is strongly corrosive, which limits the selection of materials for current collectors and battery shells [12–14]. Furthermore, the carrier ions in AlCl_3_/[EMIm]Cl IL are monovalent AlCl_4_^−^ and Al_2_Cl_7_^−^, which means that the high theoretical capacity from the three-electron transfer reactions of Al^3+^/Al cannot be fully realized [15]. Therefore, exploring chloride-free electrolyte systems that can overcome the above issues is the key to improving the performance and promoting the commercialization of AIBs.

Deep eutectic electrolytes (DEEs) are multicomponent mixtures featured by a solidification temperature considerably lower than those of their individual components and have similar properties to IL (also termed as IL analogues) [16, 17]. The chemical environments of metal ions in DEEs are totally different from those in aqueous solutions, organic solutions or water/organic mixed electrolytes, leading to significantly different electrochemical behavior [18–20]. Recently, DEEs receive extensive research interest in the field of energy storage due to the high electrochemical and thermal stability, facile synthesis, low vapor pressure, and tunable compositions [21]. Especially in the field of zinc-ion and lithium-ion batteries, eutectic electrolytes can effectively promote the reversible deposition and stripping of Zn and Li metals and suppress dendrite generation due to their unique solvation structure, resulting in enhanced cycle stability of the batteries [22–26]. However, the study on Al-based DEEs is still in the infancy stage. To date, the Al salt used for the formation of DEEs is AlCl_3_ in most research work [27–29], but AlCl_3_-based DEEs are still humidity sensitive and corrosive as AlCl_3_/[EMIm]Cl IL. More recently, our group developed a new chloride-free Al-based hydrated eutectic electrolyte composed of Al(ClO_4_)_3_·9H_2_O and succinonitrile (SN) for safe and air-stable AIBs [30]. The water molecules in this hydrated eutectic electrolyte (HEE) exhibit a similar behavior to those in water-in-salt electrolytes, predominantly existed as bound water rather than free water [31], and the unique HEE structure helps to improve ionic conductivity, reduce electrolyte viscosity and realize reversible plating/stripping of Al. Unfortunately, the used ligand SN is toxic, and the free state SN molecule in the electrolyte tend to be decomposed on metal surface [32], which may cause sustainability and stability problems of the HEEs.

The properties of HEEs vary with their compositions considerably. The selection of organic ligand in HEEs is critical, because it can directly determine the composition of the metal-containing complex, which typically associates with metal deposition and affects the interfacial chemistry between electrolyte and electrode [21]. Methylurea (MU), a urea derivative, has functional groups (C=O and − NH_2_) that can serve as both donors and acceptors of hydrogen bonds, suggesting that it is a suitable ligand candidate to form HEEs. Moreover, MU has an asymmetric molecular structure, which may further enhance the solubility of salt in the electrolyte and even lead to a lower viscosity and density as well as suppressed crystallization [23]. Additionally, MU is an economical, non-flammable and lowly toxic substance, which increases the sustainability of AIBs.

In this work, we develop a new chloride-free Al-based HEE composed of aluminum perchlorate nonahydrate and MU ligands for rechargeable Al-ion batteries. The coordination between Al^3+^ and MU triggers a deep eutectic effect, resulting in the formation of a liquid HEE from the two solid substances. The formed Al(ClO_4_)_3_·9H_2_O/MU hydrated deep eutectic electrolyte (AMHEE) is low-cost, non-corrosive, environmental benign, and good air stability. Density functional theory (DFT) reveals that both H_2_O and MU molecules coordinate with Al^3+^ in the AMHEE. With an optimized ratio of aluminum perchlorate to neutral MU (1:4), the unique solvation structure of [Al(MU)_2_(H_2_O)_4_]^3+^ can facilely realize stable and reversible reaction of Al, and the Al electrode exhibits a good cycling stability for over 150 h at 0.5 mA cm^−2^. When combining with vanadium oxide positive electrode, the Al-ion full battery delivers a high discharge capacity of 320 mAh g^−1^ with good capacity retention, and the Al^3+^ storage mechanism is revealed through in situ synchrotron radiation X-ray diffraction.

## Experimental Section

### Preparation of Hydrated Eutectic Electrolytes

The electrolytes were prepared by mixing aluminum perchlorate nonahydrate (Al(ClO_4_)_3_·9H_2_O) and methylurea (MU) with various molar ratios of 1:2, 1:4, 1:6, 1:10, and 1:14 at 60 °C for 30 min, then a clear liquid was obtained at room temperature.

### Synthesis of V_2_O_5_ Rods

V_2_O_5_ rods used for the positive electrode were synthesized according to a previous work with slight modifications. Briefly, 2 g of V_2_O_5_ powder was dissolved in 50 mL of distilled water with magnetic stirring at room temperature. Then, 10 mL of 30 wt% H_2_O_2_ was slowly added into the above solution to form a brown solution. After 1-h stirring, the solution was transferred to 100 mL Teflon-lined stainless steel reactor and heated to 180 °C for 72 h. The product was then washed by distilled water and ethanol for several times, and dried under vacuum at 80 °C overnight. Finally, the dried product was heated at 350 °C in air for 4 h to obtain V_2_O_5_ rods.

### Cell Assembly and Electrochemical Testing

For the Al-ion cell assembly, V_2_O_5_, Al foil (100 μm), Al(ClO_4_)_3_·9H_2_O/MU hydrated eutectic electrolyte (AMHEE) and glass fiber paper (Whatman GF/A) were used as positive electrode, negative electrode, electrolyte and separator, respectively. The positive electrode was made by mixing the active material, poly(vinylidene difluoride) (PVDF), super P with a weight ratio of 8:1:1 and casted on a carbon paper collector. The Al foil was polished and cleaned with ethanol before use. 2025-type coin cells were assembled in air for electrochemical measurements. Cyclic voltammetry (CV) test was implemented over the range of 0.1 ~ 2.0 V on potentiostat (Gamry, REF 600 +). Galvanostatic discharge/charge measurement was conducted within the voltage range of 0.1 ~ 1.6 V using a battery test system (LAND, CT2011A).

### Material Characterizations

X-ray diffraction (XRD, Mini Flex 600) was conducted with a Cu Kα wave from 10° to 80°. In situ synchrotron radiation X-ray diffraction (SR-XRD) was conducted through a self-designed cell equipped with an open window on the positive electrode side (Shanghai Synchrotron Radiation Facility BL14B1). Thermogravimetric analysis (TGA) of electrolytes was carried out with an STA 449 F3 at a heating rate of 5 °C min^−1^ under high purity nitrogen flow. Raman spectra were collected through Renishaw in Via Qontor. The ^17^O NMR and ^27^Al NMR spectra were recorded from 700 MHz superconducting NMR spectrometer (AVANCE NEO 700 MHz). Morphologies of V_2_O_5_ were characterized by scanning electron microscopy (SEM, Mira 3) with an energy dispersive spectroscopy (EDS). X-ray photoelectron spectroscopy (XPS) was conducted with AXIS UltraDLD spectrometer. Differential scanning calorimeter (DSC) was applied to evaluate the thermal properties of the hydrated eutectic electrolytes, in which samples were scanned from 20 to − 150 °C at a rate of 5 °C min^−1^ under nitrogen atmosphere. Time-of-flight secondary ion mass spectrometry (ToF–SIMS) in positive ion mode was performed on a TOF–SIMS 5–100 instrument (IONTOF GmbH; Muenster, Germany) equipped with a Bi cluster primary ion gun and a dual source column for depth profiling to analysis the composition of the SEI layer.

### Simulation Details

MD simulations were carried out using COMPASSII force field for electrolyte mixtures of MU, Al(ClO_4_)_3_ and H_2_O. The molecules were initially packed randomly into a periodic box and the geometries were further optimized. Five molar ratios of the electrolyte mixtures range from 2:1:9 to 14:1:9. The simulations were performed in canonical ensemble (NVT) for 20 ps and subsequent isothermal-isobaric ensemble (NPT) for another 200 ps, while the temperature at 298 K and pressure at 1 bar were controlled by Berendsen thermostat and barostat. The Ewald scheme and atom-based 15.5 Å cutoff were applied throughout all steps. All DFT calculations were performed using the Gaussian 09 package with the B3LYP level and 6–311 + G (d, p) basis set. The solvation energies of the Al^3+^-H_2_O and Al^3+^-MU were calculated according to Equation as follows:

$$E_{s} = E_{{{\text{Al}}}}^{3 + } + mE_{{{\text{H}}_{2} {\text{O}}}} + nE_{MU} {-}E_{{{\text{complex}}}}^{3 + }$$

where *E*_complex_ is the total energy, and *E*_Al_^3+^, *E*_H2O_ and *E*_MU_ are the energy for Al^3+^, H_2_O and MU molecule fragments.

## Results and Discussion

### Electrolyte Preparation and Characterizations

**Fig. 1:**
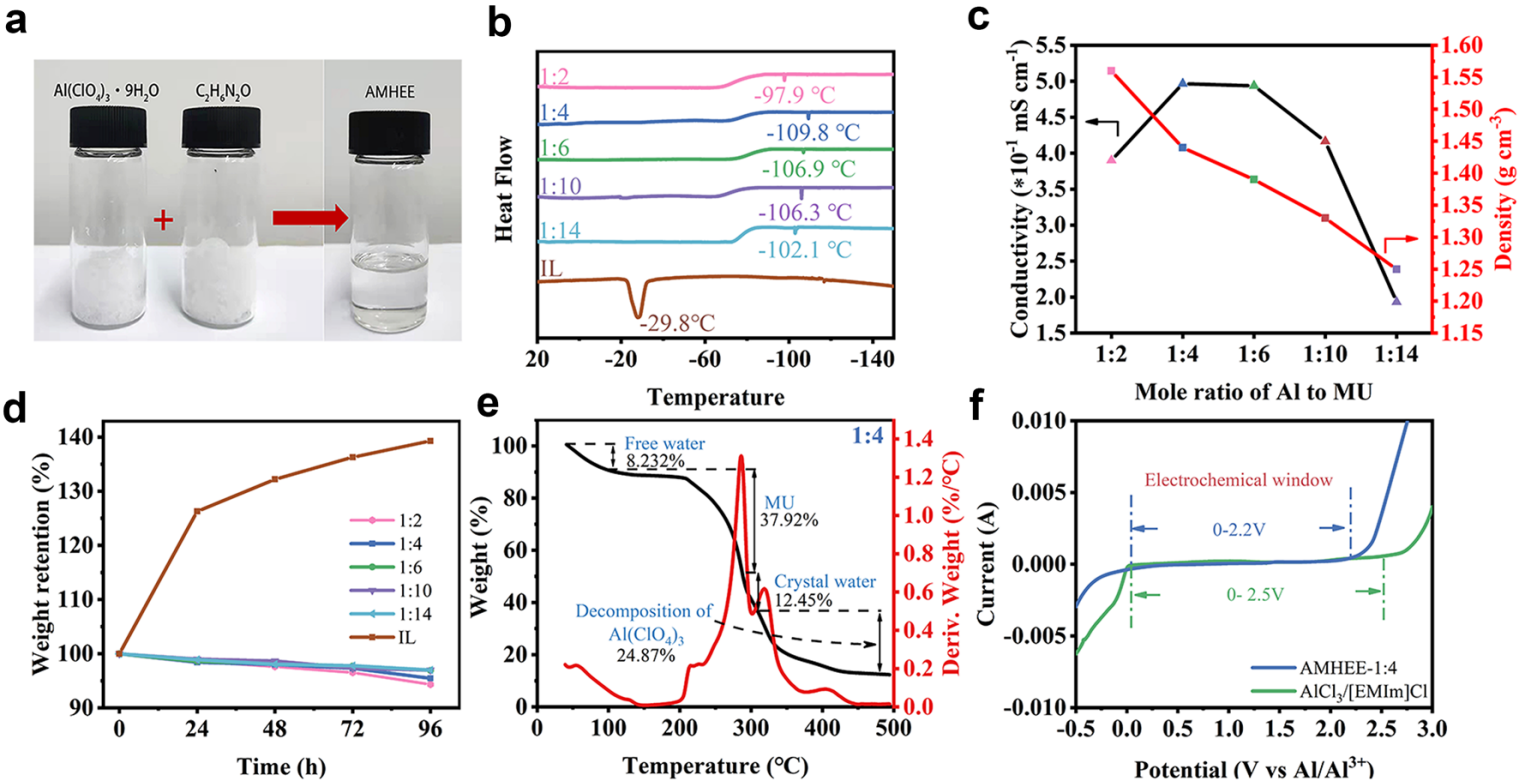
Preparation and properties of AMHEEs. **a** Synthesis of the AMHEE by mixing Al(ClO_4_)_3_·9H_2_O with MU. **b** DSC curves of the AMHEEs with different molar ratios of Al(ClO_4_)_3_·9H_2_O to MU. **c** Conductivity and density of the AMHEEs as a function of molar ratio. **d** Weight loss of AMHEEs with various molar ratios and AlCl_3_/[EMIm]Cl IL in open air (25 °C). **e** Thermogravimetric analysis of the AMHEE-1:4. **f** Electrochemical windows of the AMHEE-1:4 and AlCl_3_/[EMIm]Cl IL

MU as a low-cost and safe urea derivative has two functional groups (C=O and − NH_2_) that can serve as both acceptor and donor of hydrogen bonds. Meanwhile, Al(ClO_4_)_3_·9H_2_O, an economical Al salt with bipolar water molecule, also can serve as hydrogen bond acceptor and donor. Moreover, the interaction between the delocalized anions (ClO_4_^−^) and cations (Al^3+^) in Al(ClO_4_)_3_·9H_2_O is relatively weak [21, 33]. As a result, the liquid AMHEE can be achieved by simply mixing solid Al(ClO_4_)_3_·9H_2_O with solid MU (Fig. 1a). Noticeably, the molar ratio of Al(ClO_4_)_3_·9H_2_O to MU plays a vital role in forming the AMHEE and determines the solvation structure in the AMHEE. The molar ratio of Al(ClO_4_)_3_·9H_2_O to MU was varied from 1:2 to 1:14 to demonstrate the availability of AMHEE. The AMHEEs with ratios from 1:4 to 1:12 display homogeneous and clear liquid phase at room temperature, while the AMHEEs with molar ratios of 1:2 and 1:14 display cloudy liquid phase with observably high viscosities (Fig. S1). The formation of AMHEE can be mainly ascribed to the bipolar nature of water molecules in Al(ClO_4_)_3_·9H_2_O and functional groups (C=O and − NH_2_) in MU, and the intermolecular interaction between Al(ClO_4_)_3_·9H_2_O and MU components are stronger than those between their individual components, leading to a deep eutectic effect and resulting in formation of the AMHEE [19, 23]. Furthermore, differential scanning calorimeter (DSC) curves in Fig. 1b display that all the AMHEEs appear freezing point peaks and glass-transition temperature peaks within 20 to − 150 °C, and the freezing points of the AMHEEs vary with the molar ratio. The AMHEE with a molar ratio of 1:4 exhibits the lowest freezing point of − 109.4 °C, which is considered as its eutectic point and much lower than that of AlCl_3_/[EMIm]Cl IL (− 29.8 °C). The conductivity of the AMHEE is also related to the molar ratio. When the ratio of Al(ClO_4_)_3_·9H_2_O to MU was varied from 1:2 to 1:14, the conductivity of the AMHEE first increases to a maximum value of 0.5 mS cm^−1^ at a molar ratio of 1:4, and then gradually decreases (Fig. 1c). Meanwhile, the density of the AMHEE decreases from 1.57 to 1.25 g cm^−3^ (Fig. 1c). In addition, air compatibility of the AMHEE was evaluated. When the AMHEEs were exposed to the open air, there is little change in weight of the AMHEEs over 48 h (Fig. 1d). On the contrary, the traditional AlCl_3_/[EMIm]Cl electrolyte shows a huge increase in weight due to the strong adsorption of water. Moreover, the Raman analysis demonstrate that there is no observed change in the Raman spectra of the electrolyte before and after being exposed to air for 10 days, demonstrating its good air stability (Fig. S2). Furthermore, the AMHEE can still remain stable in liquid state without any observed phase separation after 20 days (Fig. S3). Additionally, the corrosion behavior of stainless-steel (coin cell case) and aluminum metal was evaluated. When the stainless-steel plate and aluminum foil were immersed in the AMHEE for 7 days, there is no observed change in morphology or composition on their surfaces, as evidenced by the SEM images and XRD patterns (Figs. S4–S7). These observations demonstrate that the AMHEE has good air stability and non-corrosiveness, suggesting that the batteries can be directly manufactured with common metal cases in air without any need of atmosphere control.

TGA was used to analyze the thermal stability of AMHEEs. TGA shows that the weight loss of AMHEEs can be divided into four stages (Figs. 1e and S8). First, the weight loss before 100 °C is due to the evaporation of free water, which is from the conversion of crystal water [19, 34]. The weight loss within the temperature of 100 to 300 °C is due to the evaporation of MU and the stepwise loss of crystal water. Finally, the weight loss after 300 °C is due to the decomposition of aluminum perchlorate. The result demonstrates that the water molecules in AMHEEs are in the form of both crystal and free states, and the fraction of each is given in Fig. S9. The fraction of crystal water converted to free water is close to 50% in the AMHEEs-1:2, 1:4, 1:6 and 1:10, and an almost complete conversion of crystal water to free water occurs in the AMHEE-1:14. The result suggests that the excessive MU will significantly alter the state of the original crystal water in Al(ClO_4_)_3_·9H_2_O to free water. Furthermore, the AMHEE shows a wide electrochemical window of ~ 2.2 V, which is similar to that of traditional AlCl_3_/[EMIm]Cl IL electrolyte (Fig. 1f), and this wide electrochemical window of the AMHEE is beneficial to stable charge/discharge and a possibly high operational voltage. These results demonstrate that the AMHEE is a potentially promising electrolyte for Al-ion batteries.

**Fig. 2:**
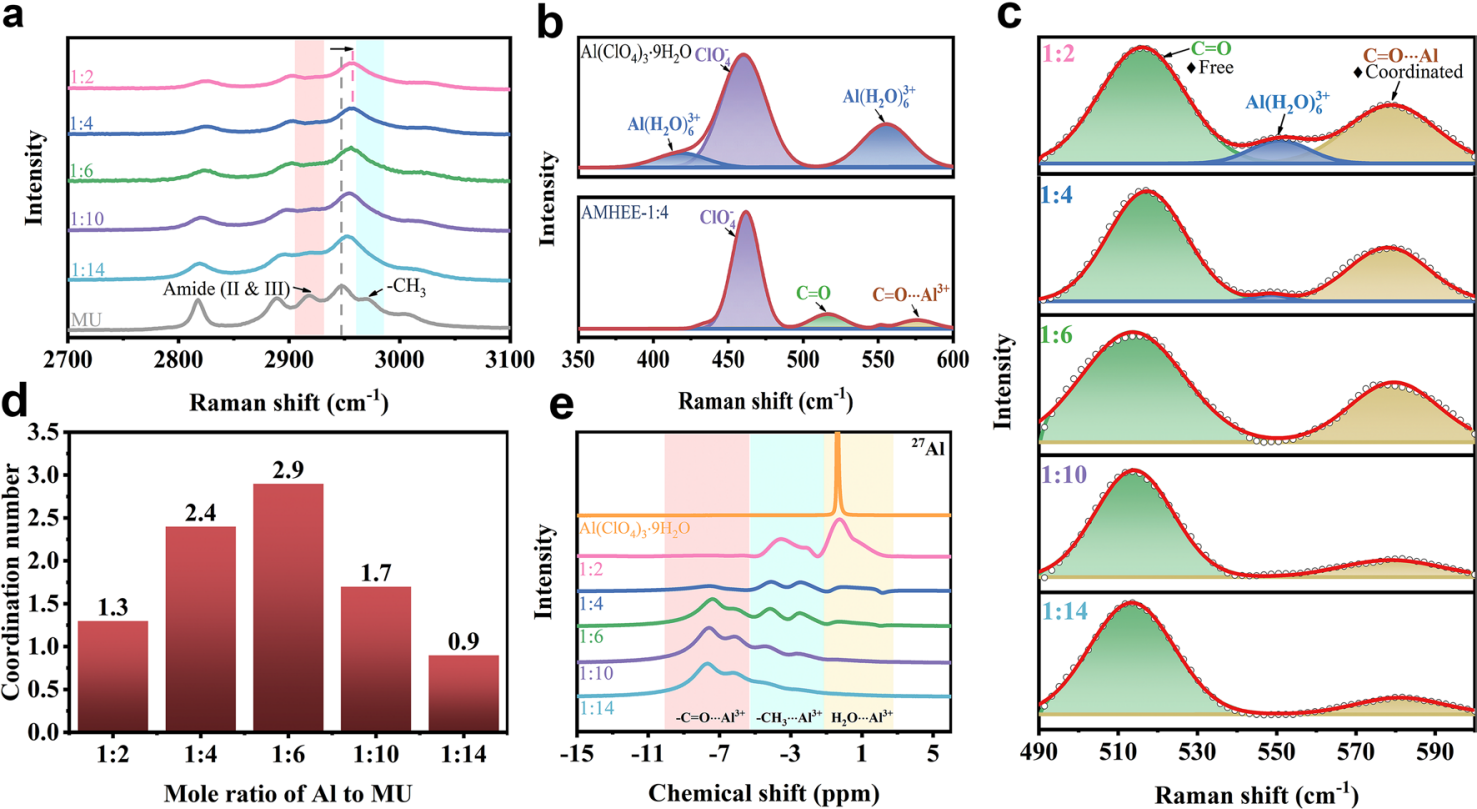
Structural characterization of the AMHEEs. Raman spectra of **a** Amide II and Amide III bands and **b** Al(ClO_4_)_3_·9H_2_O and AMHEE-1:4 and **c** C=O deformation vibration modes in ASHEEs with various concentrations. **d** Average coordination numbers of MU bound to each Al^3+^ in the AMHEEs. **e** ^27^Al NMR spectra of AMHEEs with different molar ratios

Raman spectroscopy characterization was performed to probe molecular interaction within the AMHEEs. In Fig. 2a, the characteristic peak of the AMHEEs locating at 2918 cm^−1^ is ascribed to the amide II and III bands [35], which are regarded as characteristic bands of MU molecular. The characteristic peaks of the AMHEEs centered at ~ 2948 and 2968 cm^−1^ are ascribed to the –CH_3_ asymmetric stretching vibration [35]. As the Al(ClO_4_)_3_·9H_2_O/MU ratio increase, the peaks for amide II and III bands (2918 cm^−1^) and –CH_3_ asymmetric stretching vibration (~ 2968 cm^−1^) gradually disappear, indicating that the MU in AMHEEs experiences a structure change, and the –CH_3_ in MU is involved in the coordination for the formation of AMHEE. Moreover, the –CH_3_ asymmetric stretching vibration (~ 2948 cm^−1^) of the AMHEEs gradually blue shifts with the increase in concentration of Al(ClO_4_)_3_·9H_2_O, demonstrating a stronger interaction between –CH_3_ and Al(ClO_4_)_3_·9H_2_O. In the Raman spectrum of Al(ClO_4_)_3_·9H_2_O shown in Fig. 2b, the characteristic peak at 460 cm^−1^ is assigned to ClO_4_^−^, and the peaks at 408 and 552 cm^−1^ are assigned to [Al(H_2_O)_6_]^3+^ [19]. The characteristic peaks of [Al(H_2_O)_6_]^3+^ located at 408 and 552 cm^−1^ almost disappear [19, 30]. Meanwhile, there are new characteristic peaks at 515 and 575 cm^−1^ assigning to C=O and C=O⋯Al^3+^, respectively [35]. The result reveals that the carbonyl donor group in MU can coordinate with Al^3+^ and alter the coordination between Al^3+^ and H_2_O. Furthermore, the coordination between Al^3+^ and MU is further evidenced by the C=O deformation vibration modes in Fig. 2c. The characteristic peak at ~ 515 cm^−1^ is assigned to free MU, and the other peak at 575 cm^−1^ is assigned to the coordinated MU (C=O⋯Al^3+^). According to the relative area of characteristic peaks of C=O to C=O⋯Al^3+^, the average coordination number (*N*) of MU coordinated to Al can be calculated through the following equation [36]:

1 $$N = \frac{{A_{co} /A_{t} }}{x}$$

where *A*_*co*_ is the intensity integral of the MU ligand coordinated with Al^3+^, *A*_*t*_ is the total intensity of the free and coordinated MU, and *x* is the molar fraction of Al^3+^ to total MU molecule. The calculated average coordination number of ligands is shown in Fig. 2d. With changing the molar ratio, the coordination number firstly increases and then decreases, indicating that most of the added MU is initially coordinated with Al^3+^ and then excessive MU molecule are in a free state without coordination. The average coordination number of MU in AMHEE with the ratio of 1:4 is 2.4.

^27^Al and ^17^O nuclear magnetic resonance (NMR) spectra were performed to further analyze coordination structure of the AMHEEs. The ^27^Al NMR spectra of AMHEEs in Fig. 2e exhibit three resonance ranges from 2 to − 1, − 1 to − 4, and − 4 to − 10 ppm, which are assigned to [H_2_O⋯Al^3+^], [CH_3_⋯Al^3+^] and [C=O⋯Al^3+^], respectively [37]. The NMR peak widths for the [H_2_O⋯Al^3+^] of AMHEEs are much broader than that for Al(ClO_4_)_3_·9H_2_O, which is due to the dynamic equilibrium of the chemical exchange between Al^3+^ species and crystal water or MU. Moreover, the peak for the [H_2_O⋯Al^3+^] of AMHEEs shifts to a high field and the peak intensity weakens as the MU content increases, indicating that the introduction of MU can decrease the coordination number of Al^3+^ with water. Additionally, the peak intensity for the [C=O⋯Al^3+^] increases as the Al(ClO_4_)_3_·9H_2_O /MU ratio decreases, while the peak intensity for the [CH_3_⋯Al^3+^] of AMHEE weakens, indicating that the coordination pattern of Al^3+^ with MU is dominated by [CH_3_⋯Al^3+^] bond when the MU content is low in the AMHEE, and it is converted to [C=O⋯Al^3+^] bond at a high MU content. The ^17^O NMR peaks at ~ 0 and ~ 290 ppm of the AMHEEs corresponds to H_2_O and ClO_4_^−^, respectively (Fig. S10) [19]. The peak for H_2_O almost disappears as MU content increases in AMHEE, implying that the introduction of MU can effectively reduce the activity of water in the AMHEE. The peak for ClO_4_^−^ is not much affected by the MU content, suggesting there is no coordination between ClO_4_^−^ and MU ligand.

### Molecular Dynamics Simulations

**Fig. 3:**
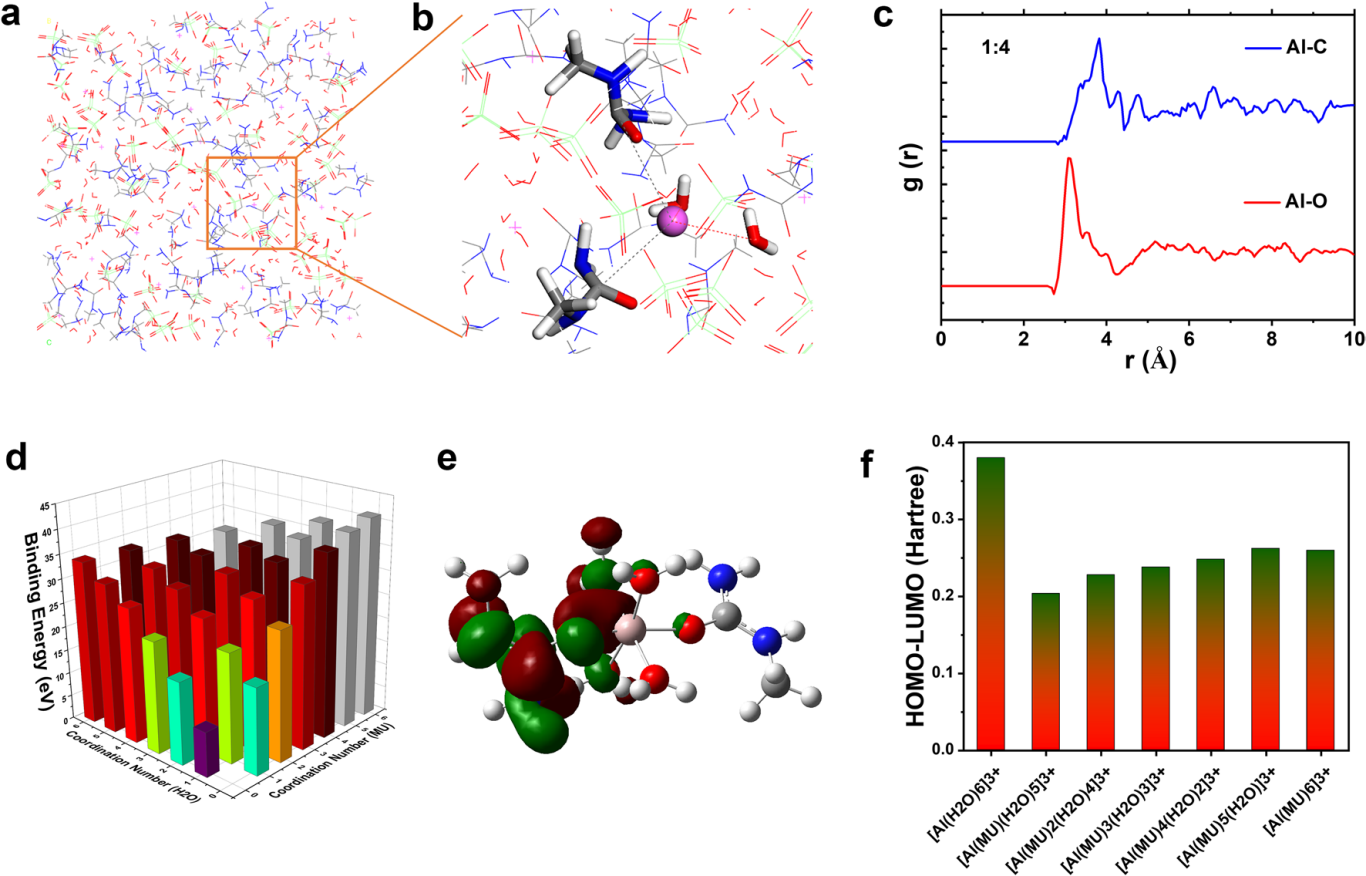
**a** Snapshot of solvation structure of Al(ClO_4_)_3_·9H_2_O/MU-1:4 from MD simulations and **b** zoomed-in area for local structure of two MU-coordinated Al-complex; **c** Radial distribution functions of Al–C and Al–O; **d** Solvation energies of Al-complex ions by DFT calculations; **e** LUMO partial charge density of [Al(MU)_2_(H_2_O)_4_]^3+^ complex; **f** Comparison of (HOMO–LUMO) energy for Al–complex with different coordination

To better understand the solvation behavior of the AMHEEs, molecular dynamics (MD) simulations of different Al(ClO_4_)_3_·9H_2_O/MU combinations were performed. The stabilized solvation structures of different molar ratios are shown in Fig. 3a and Fig. S11. The densities obtained from the simulations (Table S1) are in good agreement with the experimental findings (Fig. 1c). The porosity of AMHEEs increases remarkably from 1:2 to 1:4 while that slightly changes from 1:10 to 1:14, suggesting that MU coordination firstly replaces the denser H_2_O coordination then reaches saturation. Figure 3b is a zoomed-in area in Fig. 3a, and it is a typical local structure of Al(ClO_4_)_3_·9H_2_O/MU (1:4). It can be seen that one Al^3+^ coordinates with two H_2_O and two MU molecules, and this simulated coordination number of MU is consistent with the average number of 2.4 from the experimental measurement. Radial distribution functions (RDFs) were further used to identify the solvation structures of the AMHEEs. As shown in Figs. 3c and S12, sharp peaks of the Al–O pair and Al–C pair are recognized in all AMHEEs, corresponding to the primary solvation shells of Al^3+^ ions. When the ratio is from 1:2 to 1:6, the peak intensity of Al–O pair slightly changes but the Al–C pair shows the highest peak intensity for the combination of 1:4. When the ratio further increases, the intensity of Al–O pair decreases, suggesting that excessive MU molecules will interfere the coordination between H_2_O and Al^3+^ and even impact the Al-MU coordination. This may be the reason why the coordination number drops with the decrease in molar ratio as discussed before (Fig. 2d).

The solvation energies of various Al-complexes were also calculated by DFT (Fig. 3d and Table S2), and the optimized structures of Al-complex are shown in Fig. S13. A higher solvation energy means stronger binding among Al^3+^, H_2_O and MU segments. Obviously, the MU coordination in the solvation structure of Al^3+^ can increase the solvation energy, and a higher coordination number of MU leads to a higher solvation energy. The fully coordinated Al complex, particularly [Al(MU)_6_)]^3+^, shows the highest stability and the desolvation process during charge/discharge is the most difficult. Therefore, a medium coordination number of ≈2 for Al–MU bonding results in the best electrochemical performance. According to the frontier molecular orbital theory, the lowest unoccupied molecular orbital (LUMO) is electrophilic and electron accepting. As shown in Figs. 3e and S14, MU can only contribute to the LUMO charge density when two MU molecule coordinate with Al^3+^, and charge densities of others mainly accumulate around the H_2_O molecules. Furthermore, based on HOMO–LUMO energy gap shown in Fig. 3f, one and two MU-coordinated Al-complexes with smaller energy gaps are expected to possess larger conductivities. In addition, the analysis of Mulliken charge (Fig. S15) also shows the same trend that [Al(MU)(H_2_O)_5_]^3+^ and [Al(MU)_2_(H_2_O)_4_]^3+^ can better facilitate the electron transfer.

### Electrochemical Performance

**Fig. 4:**
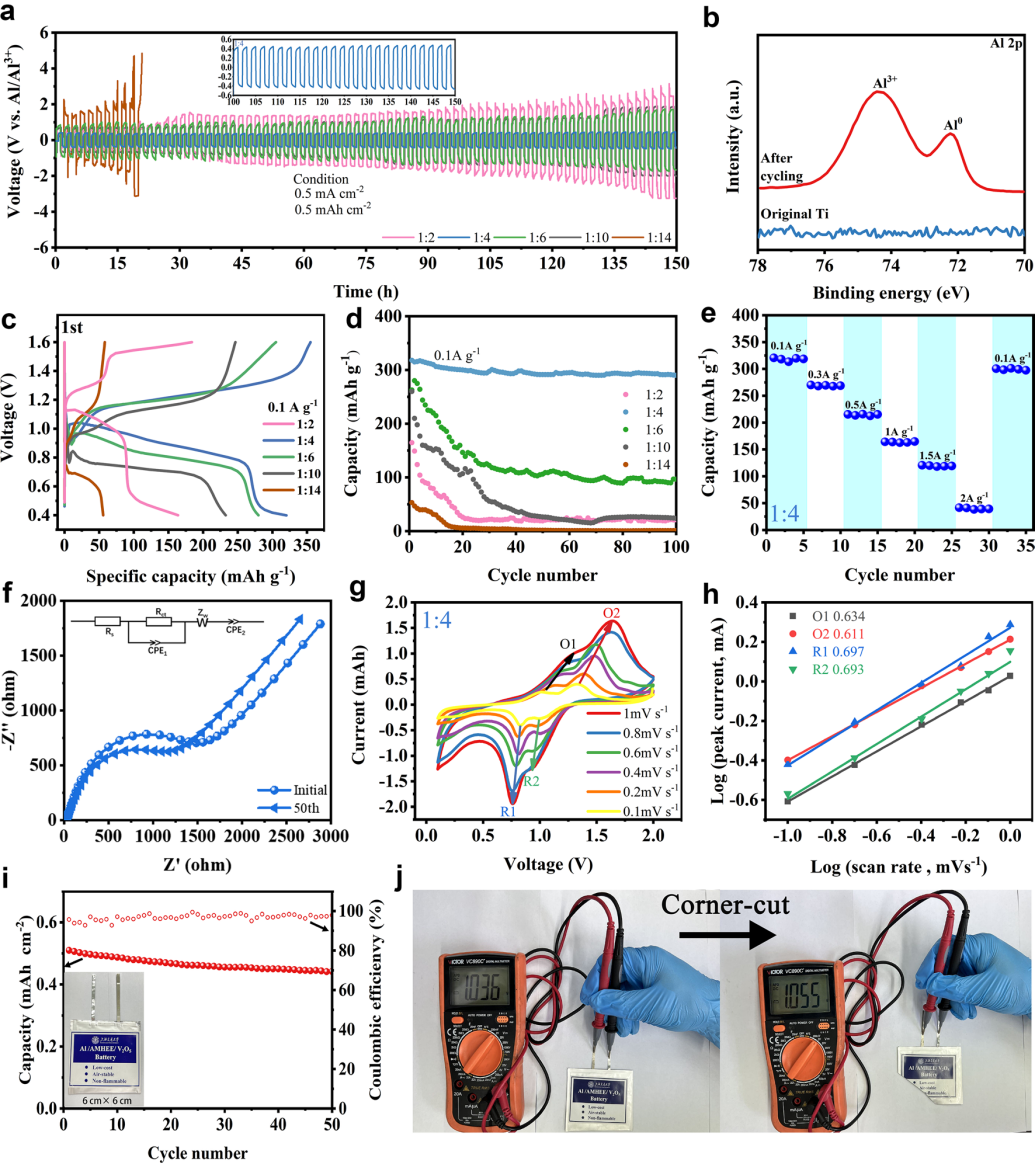
Electrochemical performance of Al cells in different AMHEEs. **a** Galvanostatic cycling curve of Al/Al symmetric cell in the AMHEE at a current density of 0.5 mA cm^−2^. **b** XPS spectra of Al 2*p* for the original and cycled Ti substrates in AMHEE. **c** Discharge/charge curves and **d** cycling performance of Al/AMHEE/V_2_O_5_ full cells. **e** Rate capability. **f** Nyquist plots. **g** CV curves at different scan rates and **h** the corresponding plots of log (*i*) vs. log (*v*) for the peak current densities of Al/AMHEE-1:4/V_2_O_5_ full cells. **i** Cycling performance of the pouch-type Al/AMHEE /V_2_O_5_ cell at 0.5 mA cm^−2^ (Inset: photo of the pouch cell (6 × 6 cm^2^). **j** OCV and corner-cut test demonstrations of the pouch AIBs

The various metal complexes in the AMHEEs usually directly associate with Al deposition and determine the interface chemistry between electrolyte and electrode [21]. Firstly, the stability of Al deposition/stripping was evaluated in different AMHEEs. The symmetric cell with AMHEE-1:4 in Fig. 4a features an extended lifespan of over 150 h with a steady voltage hysteresis at a current density of 0.5 mA cm^−2^, suggesting good cycling stability of Al electrode in AMHEE-1:4. In contrast, the symmetric cells with other AMHEEs (ratios of 1:2, 1:6, 1:10, and 1:14) exhibit increasing polarization voltages over cycling. The morphology of the Al electrode after cycling in AMHEE-1:4 was then examined, and the uneven formation of Al deposits is observed on the surface of Al foil (Fig. S16). To further evidence the plating of Al, the symmetrical cell with two pieces of titanium (Ti) foils was fabricated and cycled, and the XPS peak of the deposits at 72.3 eV reveals that metallic Al (0) was observed on the cycled Ti electrode [38–40], confirming the Al deposition in this AMHEE (Figs. 4b and S17). Energy dispersive X-Ray spectroscopy (EDS) elemental mappings display the uniform dispersion of Al, Cl, O, N and C elements on the surface, indicating there may be a SEI layer on the surface of the Al electrode after cycling (Fig. S18). The SEI layer was further evaluated by time-of-flight secondary ion mass spectrometry (TOF–SIMS). The depth profile further supports the formation of the SEI layer, which is mainly composed of various Al complexes including AlNH_2_^+^, CH_3_Al^+^, CONH_2_NHAl^+^, AlCONH_2_^+^ and Al_2_ClO_2_^+^ (Fig. S19). The three-dimensional (3D) cross section images of the Al anode after cycling reveals that the aluminum complex with ClO_4_^−^ anions are primarily located in the outermost layer of the SEI film, while the aluminum complex with MU, including AlNH_2_, CH_3_Al, CONH_2_NHAl, and AlCONH_2_, are uniformly distributed in the inner and outer layers of the SEI (Fig. S20). The 3D distribution images of the surface composition of the Al anode after cycling reveal a consistent distribution of the SEI components associated with MU and ClO_4_^−^ anions without irregular compound aggregation (Fig. S21). This confirms that the interfacial layer on the aluminum surface after cycling is a uniform SEI film rather than a residue of electrolyte aggregation.

To evaluate the compatibility and efficacy of the AMHEEs, Al-ion batteries composed of V_2_O_5_ cathode and Al anode were fabricated and tested. The as-prepared V_2_O_5_ displays a nanorod morphology (Fig. S22), and the structure is in good agreement with orthorhombic V_2_O_5_ (JCPDS #77–2418, Fig. S23), while the (001) crystal plane locating at 20.4˚ reflects the typical layered structure of V_2_O_5_ [41]. Cyclic voltammetry (CV) curves of the Al/AMHEE/V_2_O_5_ full cell with different AMHEEs electrolytes at a scan rate of 1 mV s^−1^ are shown in Figs. S24 and S25. The CV curves in the AMHEEs with ratios of 1:4, 1:6 and 1:10 display similar shape with two pairs of redox peaks. In the AMHEE-1:4, the redox peak potentials are 0.96/0.74 and 1.03/1.53 V, corresponding to different steps of the reduction (Al^3+^ insertion) and oxidation (Al^3+^ extraction) processes of V_2_O_5_ [42]. Notably, the cell with AMHEE-1:4 electrolyte displays the most positive peak potential and the largest peak area, suggesting the highest operational voltage and largest capacity. Typical galvanostatic charge/discharge tests were carried out between 0.4 and 1.6 V (Figs. 4c and S26). As the Al(ClO_4_)_3_·9H_2_O/MU ratio decreases, the Al/AMHEE/V_2_O_5_ full cells exhibit a significant decrease in discharge capacity with a drop in discharge voltage plateau. The Al/AMHEE-1:4/V_2_O_5_ full cell delivers the largest reversible specific capacity of 320 mAh g^−1^. It is necessary to mention that the carbon paper current collector contributes little to the specific capacity (Fig. S27). The CV and charge/discharge results demonstrate that the molar ratio of Al(ClO_4_)_3_·9H_2_O to MU plays a vital role in determining the electrochemical performances of the Al-ion batteries, and the molar ratio of 1:4 contributes to the best electrochemical performance. This is mainly because the difference in molar ratio lead to different coordination between Al(ClO_4_)_3_·9H_2_O and MU, and the optimized Al complex [Al(MU)_2_(H_2_O)_4_]^3+^ in the AMHEE-1:4 together with the highest conductivity contributes to the maximum Al^3+^ storage.

Cycling stability of the Al-ion batteries with different AMHEEs was also evaluated. The Al/AMHEE/V_2_O_5_ full cell can still deliver a specific capacity of 302 mAh g^−1^ at 0.1 A g^−1^ after 100 cycles (Fig. 4d). In sharp contrast, the Al-ion batteries using the AMHEEs with other ratios show quick decay in capacity (Fig. S28). Namely, the Al/AMHEE/V_2_O_5_ full cell shows the best cycling stability and reveals a good compatibility of the AMHEE-1:4 with V_2_O_5_ positive electrode, which can be explained by high conductivity and the unique solvation structure of the [Al(MU)_2_(H_2_O)_4_]^3+^ complex in AMHEE-1:4. Specifically, the Raman data and calculations above show that when the Al(ClO_4_)_3_·9H_2_O:MU molar ratio is lower than 1:4, a large amount of highly coordinated Al^3+^-MU complexes and free MU molecules are present in the AMHEE, and the higher desolvation energy of the highly coordinated Al^3+^-MU complexes inevitably cause cation-ligand co-intercalation, leading to structure destruction of V_2_O_5_. When the Al(ClO_4_)_3_·9H_2_O:MU molar ratio is higher than 1:4, a large number of free MU molecule in the AMHEE may lead to side reactions at a high voltage, resulting in capacity loss. The rate performance of the Al/AMHEE-1:4/V_2_O_5_ cell is demonstrated in Fig. 4e, and the specific discharge capacities are 320, 268, 215, 164, 120 and 41 mAh g^−1^ at the current densities of 0.1, 0.3, 0.5, 1.0, 1.5, and 2.0 A g^−1^, respectively. The remarkable electrochemical performance of AIBs based on AMHEE-1:4 is superior to the case of previously reported V_2_O_5_-based AIBs (Table S3). Electrochemical impedance spectroscopy (EIS) of the Al/AMHEE-1:4/V_2_O_5_ cell after cycling shows that the solution resistance (*R*_s_) exhibits a negligible variance compared to initial battery, but the charge transfer resistance (*R*_ct_) decreases from initial *R*_ct_ = 924.7 Ω to *R*_ct_ = 653.2 Ω (Fig. 4f), indicating a significantly improved kinetics during the cycling process. CV was then conducted at various scan rates to reveal the kinetics of the Al/AMHEE-1:4/V_2_O_5_ full cell (Fig. 4g). The relationship between peak current (*i*) and scan rate (*v*) can be written as follows [43]:

2 $$i = av^{b}$$

where *a* and *b* are constants. The *b* value can be used to judge the dominant kinetics of charge and discharge processes. Recalling that b = 1 corresponds to a capacitive-limited process, whereas *b* = 0.5 exclusively indicates a diffusion-controlled process [44]. According to the slopes of the log(*i*) vs. log(*v*) plots in Fig. 4h, the calculated b values corresponding to peaks of O1, O2, R1, and R2 are 0.634, 0.611, 0.697, and 0.693, respectively. This implies that the corresponding redox reactions are dominated by both ionic diffusion and pseudo capacitance.

The good electrochemical performance of the Al/AMHEE-1:4/V_2_O_5_ coin cells was transferred to pouch cells. The assembled Al/AMHEE-1:4/V_2_O_5_ pouch cell (6 cm × 6 cm) displays an open circuit voltage of ~ 1.2 V with a good self-discharge performance, which is evidenced by the invariable open circuit voltage over 24 h (Fig. S29). Moreover, the Al/AMHEE-1:4/V_2_O_5_ pouch cell can deliver a capacity of 0.51 mAh cm^−2^ with a capacity retention of 92.1% at a current density of 0.5 mA cm^−2^ after 50 cycles (Fig. 4i). Additionally, the pouch cell was subjected to corner-cut test, and the open circuit voltage of the pouch cell after cutting can still remain invariable (Fig. 4j), demonstrating the good safety and air stability of Al/AMHEE/V_2_O_5_ cell.

### Energy Storage Mechanism

**Fig. 5:**
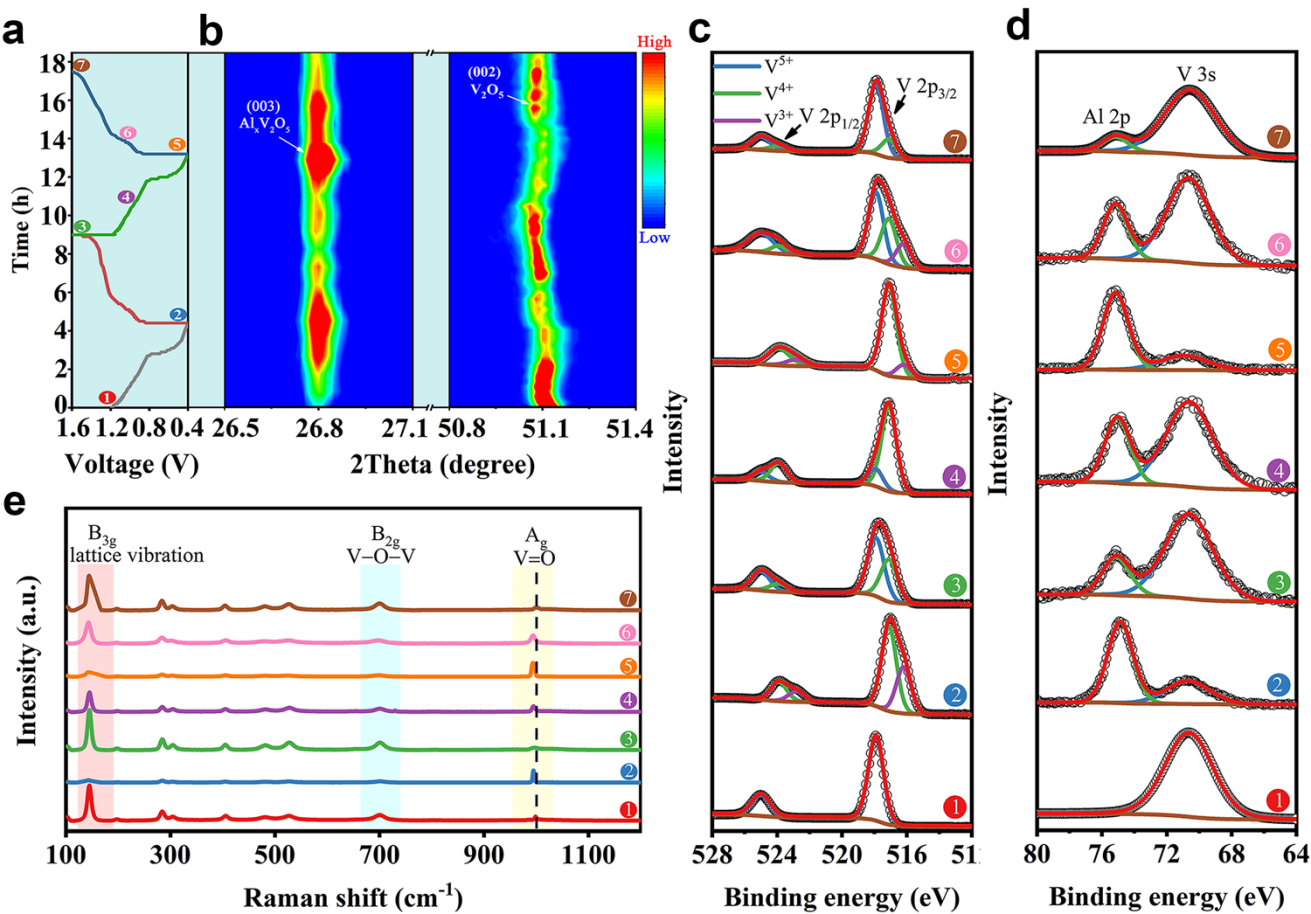
**a** Galvanostatic charge/discharge curves of Al/AMHEE-1:4/V_2_O_5_ full cell during in situ synchrotron radiation X-ray diffraction test and **b** synchrotron radiation X-ray diffraction patterns corresponding 2D color-filled contour plot. **c** V 2*p* XPS, **d** Al 2*p* XPS, and **e** Raman spectra of V_2_O_5_ electrodes at different discharge/charge states

The charge/discharge mechanism of the Al-ion battery in the AMHEE was studied. The structural evolution of V_2_O_5_ was monitored and discussed to reveal the energy storage mechanism. Firstly, the change in structure can be visibly observed. The positive electrode is initially yellow, which is the typical color of V^5+^ compound. Then, the color of the electrode becomes dark after discharging, indicating the reduction of V_2_O_5_ (Fig. S30). The color of the electrode reverts to yellow after complete charging, confirming the reversible reaction of V_2_O_5_ during the charge/discharge process. More importantly, in situ synchrotron radiation X-ray diffraction (SRXRD) was conducted to monitor the structural evolution of V_2_O_5_ during charge/discharge process. The phase changes of V_2_O_5_ during Al^3+^ intercalation and deintercalation can be fingerprinted by identifying the disappeared and newly emerged peaks of SRXRD patterns, and the reversible phase evolution associated with the reversible charge/discharge processes of V_2_O_5_ is in situ observed in Fig. 5a, b. During the first discharge process, a new phase appeared at 26.8˚, corresponding to the formation of aluminum vanadium oxide Al_x_V_2_O_5_, and the peak intensity gradually increased with the drop in discharge voltage. The intensity of (002) peak of layered V_2_O_5_ located at 51.1˚ gradually decreases along with the drop in discharge voltage. At the end of the discharge, the (002) peak of V_2_O_5_ disappeared, while the (003) peak of Al_x_V_2_O_5_ displays the highest intensity. During the charge process, the disappeared (002) peak of V_2_O_5_ appears, and the intensity gradually increases with the increase in charge voltage. Meanwhile, the intensity of aluminum vanadium oxide gradually decreases until disappears at the end of the charge process, indicating that the original layered V_2_O_5_ gradually restores by the extraction of Al^3+^ during the charging process. In the subsequent discharge/charge processes, the obvious intensity changes of diffraction patterns of V_2_O_5_ and aluminum vanadium oxide can also be observed, and the change of the diffraction pattern is consistent with that of the previous cycle, demonstrating that the reversible Al^3+^ intercalation/deintercalation process.

Ex situ XPS analysis was performed to further probe the surface chemistry change of the V_2_O_5_ electrode at different charge/discharge states marked in Fig. 5a. The V 2*p* spectrum was fitted to the splitting peaks. The peaks of V 2*p*_3/2_ at 517.9 eV, 517.0 eV and 516.2 eV correspond to V^5+^, V^4+^ and V^3+^, respectively (Fig. 5c) [42]. The V 2*p* spectrum show that the original V_2_O_5_ exhibits only V^5+^ (Curve 1). When first discharging to 0.4 V, the peak of V^5+^ almost disappears whereas the peaks of V^4+^ and V^3+^ appear (Curve 2), indicating the reduction of V_2_O_5_. Then first charging to 1.6 V, the peak of V^5+^ reappears with the presence of the V^4+^ peak whereas the V^3+^ peak disappears (Curve 3). Notably, the charge process cannot convert all V^4+^ back to V^5+^, indicating the incomplete oxidation of aluminum vanadium oxide. When the battery was discharged again to 1.0 V, the peaks of V^5+^ and V^4+^ co-exist without V^3+^, but the peak intensity of V^4+^ is much greater than that of V^5+^ (Curve 4), demonstrating that V^5+^ is gradually converted to V^4+^ and no disproportionation reaction occurs during this discharge process. When further discharging to 0.4 V, the peak of V^3+^ appears with the presence of the V^4+^ peak whereas the V^5+^ peak disappears (Curve 5). It is worth noting that when charging to 1 V, the signal of V^5+^, V^4+^ and V^3+^ are all present (Curve 6), which may be due to the disproportionate reaction of V_2_O_5_ [42]. Finally, when charging to 1.6 V during the second charge process, the valence of vanadium is same as that for the first being charged to 1.6 V (Curve 7). The Al 2*p* spectra of the V_2_O_5_ positive electrode at different charged/discharged states were analyzed to further confirm the Al^3+^ storage mechanism of in V_2_O_5_ (Fig. 5d). As expected, there is no Al signal detected in the positive electrode at the initial state (Curve 1). The Al 2*p* peak at 74.2 eV was observed after first discharge. Apparently, the intensity ratio of the Al 2*p* peak to the V 3* s* peak varies with the change in the charge/discharge voltage, and the peak intensity ratio reaches the maximum at the fully discharged state (Curve 2 and 5), the minimum at the fully charged state (Curve 3 and 7), and the intermediate value at the partially charged/discharged state (Curve 4 and 6). Furthermore, the EDS mappings demonstrate that of the V_2_O_5_ displays a strong Al signal when discharging at 0.4 V and a weak Al signal when charging at 1.6 V (Fig. S31). These results clearly evidence that reversible reaction between Al^3+^ and V_2_O_5_ during the discharging/charging process.

Figure 5e shows the Raman spectra of V_2_O_5_ electrode at different states. At the initial state of V_2_O_5_ (Point 1), the narrow peak at 997 cm^−1^ is attributed to the shortest vanadium–oxygen bond (vanadyl V=O) [45], while the peak at 706 cm^−1^ is assigned to the doubly coordinated oxygen (V − O − V) stretching mode resulting from corner-shared oxygen common to two pyramids, and the peak at 143 cm^−1^ is assigned to the lattice vibration, which are typical for the layered V_2_O_5_ [41]. When first discharging to 0.4 V (Point 2), the peaks at 143 and 706 cm^−1^ almost disappear and the intensity of the peak at 997 cm^−1^ increases, indicating the transformation of V_2_O_5_. Then charging to 1.6 V, the peaks at 143 and 706 cm^−1^ reappears, and the intensity of the peak at 997 cm^−1^ decreases, indicating the V_2_O_5_ structure is recovered. During the second discharge/charge process, the intensities of the peaks at 143, 706, and 997 cm^−1^ vary with the change in charge/discharge voltage, which is consistent with those of the first cycle. Furthermore, the Raman peak of vanadium–oxygen bond at 997 cm^−1^ for initial V_2_O_5_ (Point 1) shifts to 992 cm^−1^ when discharging to 0.4 V (Point 2 and Point 5), which is attributed to the elongation of vanadyl bonds caused by the insertion of Al^3+^ into V_2_O_5_. Moreover, the corresponding peak blue shifts when charging to 1.6 V (Point 3 and Point 7), meaning a strengthened vanadium–oxygen bond caused by the extraction of Al^3+^ from V_2_O_5_. These reveal that the layered structure of V_2_O_5_ experienced a periodic reversible change during the charging/discharging process.

Through the analysis of a series of in/ex situ characterizations above, the Al^3+^ storage mechanism can be proposed that: the Al^3+^ ions reacted with V_2_O_5_ to form Al_x_V_2_O_5_ during the discharge process, and the Al^3+^ ions are extracted from Al_x_V_2_O_5_ to recover V_2_O_5_ during the charge process. Accordingly, the reversible charge/discharge reaction is summarized as follows:

$${\text{V}}_{{2}} {\text{O}}_{{5}} + x{\text{Al}}^{3 + } + 3xe^{ - } \underset{{{\text{Charge}}}}{\overset{{{\text{Discharge}}}}{\longleftrightarrow}}{\text{Al}}_{{\text{X}}} {\text{V}}_{2} {\text{O}}_{5}$$

It is noteworthy that the mechanism of Al^3+^ inserted into V_2_O_5_ differs from those of other aluminum battery systems. In the aluminum batteries using AlCl_3_/[EMIm]Cl electrolyte, the carrier ions are AlCl_4_^−^ and Al_2_Cl_7_^−^ complex in the electrolyte. The strong interaction between the anions and cations makes it difficult for the carrier ions to desolvation and transform into Al^3+^ during the insertion process into V_2_O_5_. Moreover, the large size of the carrier ions cause the difficulty in the insertion into V_2_O_5_. In the aluminum batteries using aqueous Al(OTF)_3_ electrolytes, it’s reported that interaction of Al^3+^ in V_2_O_5_ was not found [41], which was explained by the difficulty in dissociation of complex ions of Al(OTF)_2_^+^ [41, 46]. In this study, the unique solvation structure of Al^3+^ in the AMHEE has a low desolvation energy to facilitate the interaction of Al^3+^ in V_2_O_5_, which was supported by the clear evidence mentioned above.

## Conclusions

We propose a novel hydrated eutectic electrolyte composed of aluminum perchlorate and neutral methylurea, which can realize reversible deposition/stripping of Al with three-electron transfer. The AMHEE electrolyte features facile formulation, low cost, non-flammability and air compatibility. With an optimized ratio of aluminum perchlorate to neutral methylurea (1:4), the AMHEE electrolyte presents a unique solvation structure and a high conductivity. Correspondingly, the Al//V_2_O_5_ full cell with AMHEE delivers a high capacity of 320 mAh g^−1^ with good cycling stability, and the corresponding pouch cell also delivers high specific discharge capacity with good capacity retention. Furthermore, DFT calculation confirms AMHEE-1:4 has an enhanced ion transport kinetics which benefits to the unique [Al(MU)_2_(H_2_O)_4_]^3+^ carrier ion. This work proposes a new way of developing low-cost, non-corrosive, and non-flammable electrolytes for large-scale energy storage.

### Supplementary Information

Below is the link to the electronic supplementary material.

Supplementary file 1 (PDF 8823 KB)

## References

[1] Deng J, Bae C, Denlinger A, Miller T (2020). Electric vehicles batteries: requirements and challenges. Joule.

[2] Albertus P, Babinec S, Litzelman S, Newman A (2018). Status and challenges in enabling the lithium metal electrode for high-energy and low-cost rechargeable batteries. Nat. Energy.

[3] Yang H, Li H, Li J, Sun Z, He K (2019). The rechargeable aluminum battery: opportunities and challenges. Angew. Chem. Int. Ed..

[4] Liang Y, Dong H, Aurbach D, Yao Y (2020). Current status and future directions of multivalent metal-ion batteries. Nat. Energy.

[5] Wang F, Jiang M, Zhao T, Meng P, Ren J (2022). Atomically dispersed iron active sites promoting reversible redox kinetics and suppressing shuttle effect in aluminum–sulfur batteries. Nano-Micro Lett..

[6] Sun H, Wang W, Yu Z, Yuan Y, Wang S (2015). A new aluminium-ion battery with high voltage, high safety and low cost. Chem. Commun..

[7] Zhang X, Jiao S, Tu J, Song W-L, Xiao X (2019). Rechargeable ultrahigh-capacity tellurium–aluminum batteries. Energy Environ. Sci..

[8] Lin M-C, Gong M, Lu B, Wu Y, Wang D-Y (2015). An ultrafast rechargeable aluminium-ion battery. Nature.

[9] Han X, Bai Y, Zhao R, Li Y, Wu F (2022). Electrolytes for rechargeable aluminum batteries. Prog. Mater. Sci..

[10] Zhu N, Zhang K, Wu F, Bai Y, Wu C (2021). Ionic liquid-based electrolytes for aluminum/magnesium/sodium-ion batteries. Energy Mater. Adv..

[11] Wang S, Jiao S, Wang J, Chen H-S, Tian D (2017). High-performance aluminum-ion battery with Cus@C microsphere composite cathode. ACS Nano.

[12] Faegh E, Ng B, Hayman D, Mustain WE (2021). Practical assessment of the performance of aluminium battery technologies. Nat. Energy.

[13] Kumar S, Rama P, Yang G, Lieu WY, Chinnadurai D (2022). Additive-driven interfacial engineering of aluminum metal anode for ultralong cycling life. Nano-Micro Lett..

[14] Yu Z, Jiao S, Li S, Chen X, Song W-L (2019). Flexible stable solid-state al-ion batteries. Adv. Funct. Mater..

[15] Zhang Y, Liu S, Ji Y, Ma J, Yu H (2018). Emerging nonaqueous aluminum-ion batteries: challenges, status, and perspectives. Adv. Mater..

[16] Zhang C, Zhang L, Yu G (2020). Eutectic electrolytes as a promising platform for next-generation electrochemical energy storage. Acc. Chem. Res..

[17] Wu J, Liang Q, Yu X, Lü Q-F, Ma L (2021). Deep eutectic solvents for boosting electrochemical energy storage and conversion: A review and perspective. Adv. Funct. Mater..

[18] Zhu Y, Guo X, Lei Y, Wang W, Emwas A-H (2022). Hydrated eutectic electrolytes for high-performance mg-ion batteries. Energy Environ. Sci..

[19] Yang W, Du X, Zhao J, Chen Z, Li J (2020). Hydrated eutectic electrolytes with ligand-oriented solvation shells for long-cycling zinc-organic batteries. Joule.

[20] Dou Q, Yao N, Pang WK, Park Y, Xiong P (2022). Unveiling solvation structure and desolvation dynamics of hybrid electrolytes for ultralong cyclability and facile kinetics of Zn–Al alloy anodes. Energy Environ. Sci..

[21] Geng L, Wang X, Han K, Hu P, Zhou L (2022). Eutectic electrolytes in advanced metal-ion batteries. ACS Energy Lett..

[22] Geng L, Meng J, Wang X, Han C, Han K (2022). Eutectic electrolyte with unique solvation structure for high-performance zinc-ion batteries. Angew. Chem. Int. Ed..

[23] Lin R, Ke C, Chen J, Liu S, Wang J (2022). Asymmetric donor-acceptor molecule-regulated core-shell-solvation electrolyte for high-voltage aqueous batteries. Joule.

[24] Lu X, Hansen EJ, He G, Liu J (2022). Eutectic electrolytes chemistry for rechargeable zn batteries. Small.

[25] Song J, Si Y, Guo W, Wang D, Fu Y (2021). Organosulfide-based deep eutectic electrolyte for lithium batteries. Angew. Chem. Int. Ed..

[26] Xiong P, Zhang Y, Zhang J, Baek SH, Zeng L (2022). Recent progress of artificial interfacial layers in aqueous zn metal batteries. EnergyChem.

[27] Angell M, Pan C-J, Rong Y, Yuan C, Lin M-C (2017). High coulombic efficiency aluminum-ion battery using an alcl_3_-urea ionic liquid analog electrolyte. Proc. Natl. Acad. Sci..

[28] Angell M, Zhu G, Lin M-C, Rong Y, Dai H (2020). Ionic liquid analogs of alcl_3_ with urea derivatives as electrolytes for aluminum batteries. Adv. Funct. Mater..

[29] Fang Y, Yoshii K, Jiang X, Sun X-G, Tsuda T (2015). An AlCl_3_ based ionic liquid with a neutral substituted pyridine ligand for electrochemical deposition of aluminum. Electrochim. Acta.

[30] Meng P, Huang J, Yang Z, Wang F, Lv T (2022). A low-cost and air-stable rechargeable aluminum-ion battery. Adv. Mater..

[31] Liu X, Yu Z, Sarnello E, Qian K, Seifert S (2021). Microscopic understanding of the ionic networks of “water-in-salt” electrolytes. Energy Mater. Adv..

[32] Hu Z, Xian F, Guo Z, Lu C, Du X (2020). Nonflammable nitrile deep eutectic electrolyte enables high-voltage lithium metal batteries. Chem. Mater..

[33] Tian Z, Zou Y, Liu G, Wang Y, Yin J (2022). Electrolyte solvation structure design for sodium ion batteries. Adv. Sci..

[34] Hammond OS, Bowron DT, Edler KJ (2017). The effect of water upon deep eutectic solvent nanostructure: An unusual transition from ionic mixture to aqueous solution. Angew. Chem. Int. Ed..

[35] Saito Y, Machida K, Uno T (1975). Vibrational spectra of methylurea. Spectrochim. Acta A.

[36] Ghazvini MS, Pulletikurthi G, Lahiri A, Endres F (2016). Electrochemical and spectroscopic studies of zinc acetate in 1-ethyl-3-methylimidazolium acetate for zinc electrodeposition. ChemElectroChem.

[37] Haouas M, Taulelle F, Martineau C (2016). Recent advances in application of ^27^al nmr spectroscopy to materials science. Prog. Nucl. Mag. Res. Sp..

[38] Yan C, Lv C, Jia B-E, Zhong L, Cao X (2022). Reversible al metal anodes enabled by amorphization for aqueous aluminum batteries. J. Am. Chem. Soc..

[39] Sherwood PMA (1998). Introduction to studies of aluminum and its compounds by XPS. Surf. Sci. Spectra..

[40] Bou M, Martin JM, Le Mogne T, Vovelle L (1991). Chemistry of the interface between aluminium and polyethyleneterephthalate by XPS. Appl. Surf. Sci..

[41] Zhao Q, Liu L, Yin J, Zheng J, Zhang D (2020). Proton intercalation/de-intercalation dynamics in vanadium oxides for aqueous aluminum electrochemical cells. Angew. Chem. Int. Ed..

[42] Gu S, Wang H, Wu C, Bai Y, Li H (2017). Confirming reversible Al^3+^ storage mechanism through intercalation of Al^3+^ into V_2_O_5_ nanowires in a rechargeable aluminum battery. Energy Storage Mater..

[43] Yoo D-J, Heeney M, Glöcklhofer F, Choi JW (2021). Tetradiketone macrocycle for divalent aluminium ion batteries. Nat. Commun..

[44] Wang S, Huang S, Yao M, Zhang Y, Niu Z (2020). Engineering active sites of polyaniline for alcl_2_^+^ storage in an aluminum-ion battery. Angew. Chem. Int. Ed..

[45] Ramana CV, Smith RJ, Hussain OM, Massot M, Julien CM (2005). Surface analysis of pulsed laser-deposited v_2_o_5_ thin films and their lithium intercalated products studied by raman spectroscopy. Surf. Interface Anal..

[46] Li Y, Liu L, Lu Y, Shi R, Ma Y (2021). High-energy-density quinone-based electrodes with [Al(OTF)]^2+^ storage mechanism for rechargeable aqueous aluminum batteries. Adv. Funct. Mater..

