# Supplementary material for: Air-Stable Binary Hydrated Eutectic Electrolytes with Unique Solvation Structure for Rechargeable Aluminum-Ion Batteries
Source: Nanomicro Lett. 2023 Jul 29;15:188. doi: 10.1007/s40820-023-01160-z (PMC10387020; doi:10.1007/s40820-023-01160-z)
Supplement: Supplementary file 1 — (PDF 8823 KB) [file 40820_2023_1160_MOESM1_ESM.pdf]

Supporting Information for

Air-Stable Binary Hydrated Eutectic Electrolytes with Unique Solvation Structure for Rechargeable Aluminum-Ion Batteries

Pengyu Meng[1, #], Jian Huang[2, #], Zhaohui Yang[1], Min Jiang[1], Yibo Wang[1], Wei Zhang[3], Jiao Zhang[1], Baode Sun[1], Chaopeng Fu[1, *]

[1] School of Materials Science and Engineering, Shanghai Jiao Tong University, Shanghai 200240, P. R. China

[2] State Key Laboratory of High Performance Ceramics and Superfine Microstructures, Shanghai Institute of Ceramics, Chinese Academy of Sciences, Shanghai 200050, P. R. China

[3] Advanced Technology Institute, University of Surrey, Guildford, Surrey GU2 7XH, UK

[#] Pengyu Meng and Jian Huang contributed equally to this work.

*Corresponding author. E-mail: chaopengfu@sjtu.edu.cn (C. Fu)

Supplementary Figures

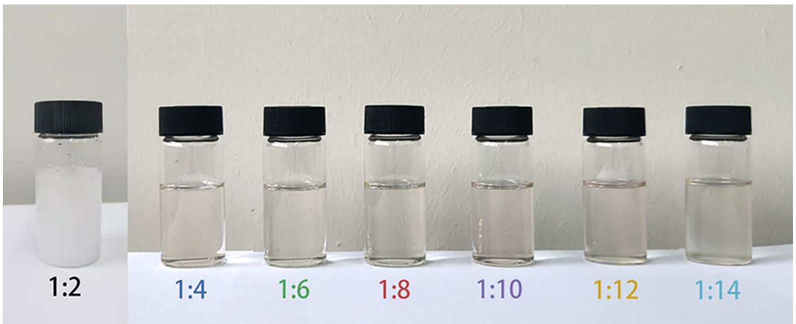

Fig. S1 Digital photos of $Al(ClO_4)_3 \cdot 9H_2O$/MU mixtures with different molar ratios (1:2, 1:4, 1:6, 1:8, 1:10, 1:12 and 1:14)

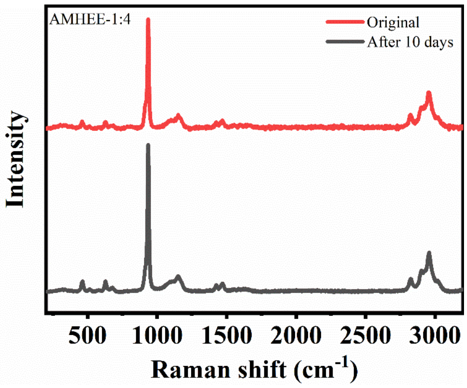

Fig. S2 Raman spectra of the electrolyte before and after being exposed to air for 10 days

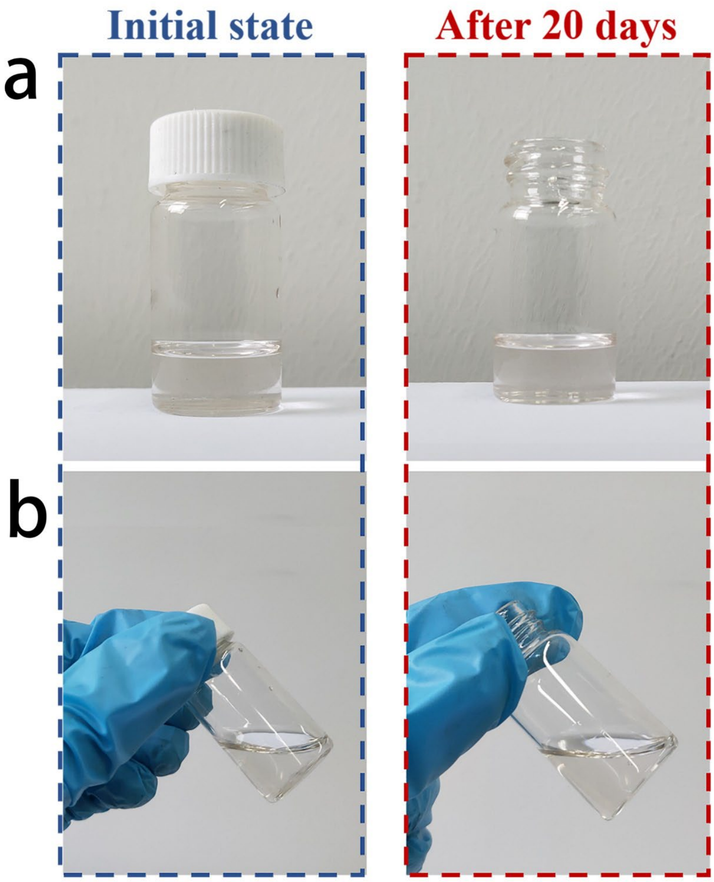

Fig. S3 (**a**) Air stability of AMHEE-1:4 in the air (25 ℃) and (**b**) fluidity comparison of the samples after 20 days

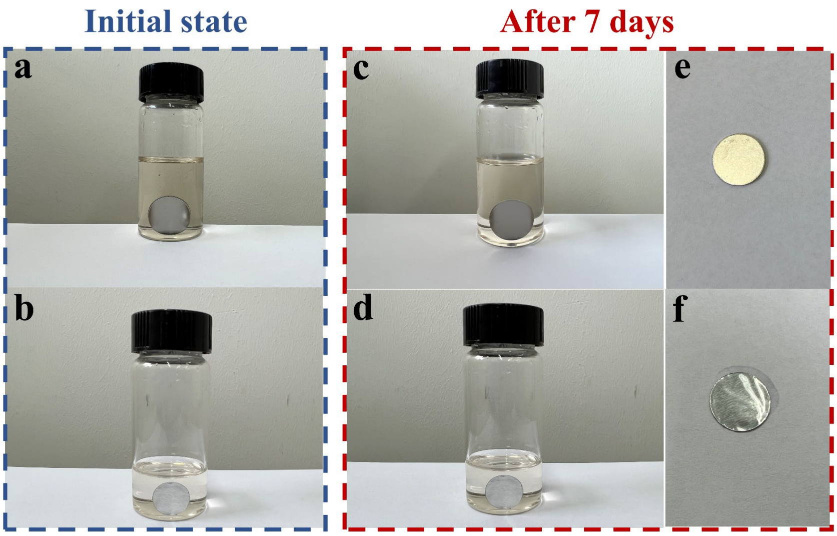

Fig. S4 Corrosive properties of the AMHEE. Initial (**a**) stainless-steel sheet and (**b**) aluminum foil. (**c**) Stainless-steel sheet and (**d**) aluminum foil submerged in the AMHEE for a duration of 7 days. (**e**) Stainless-steel sheet and (**f**) aluminum foil after removal from the AMHEE

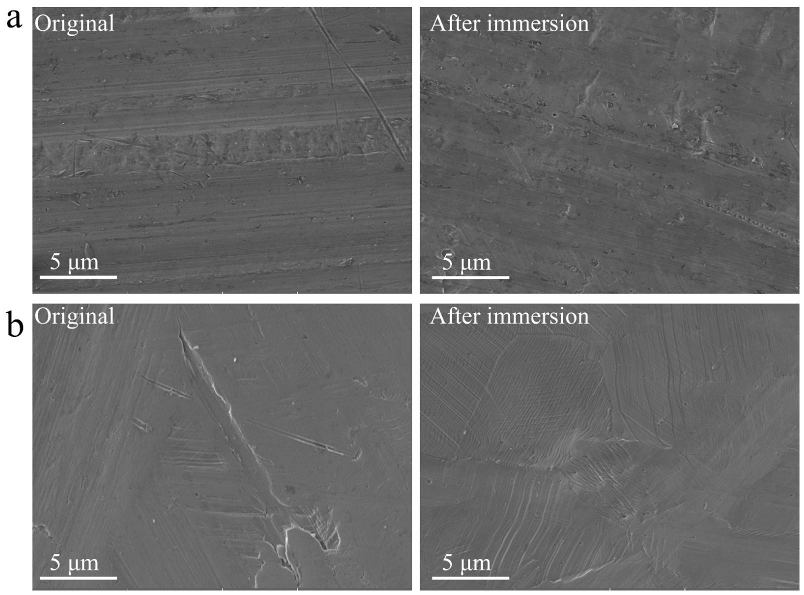

Fig. S5 SEM of the (**a**) aluminum foil and (**b**) stainless steel before and after immersion in AMHEE

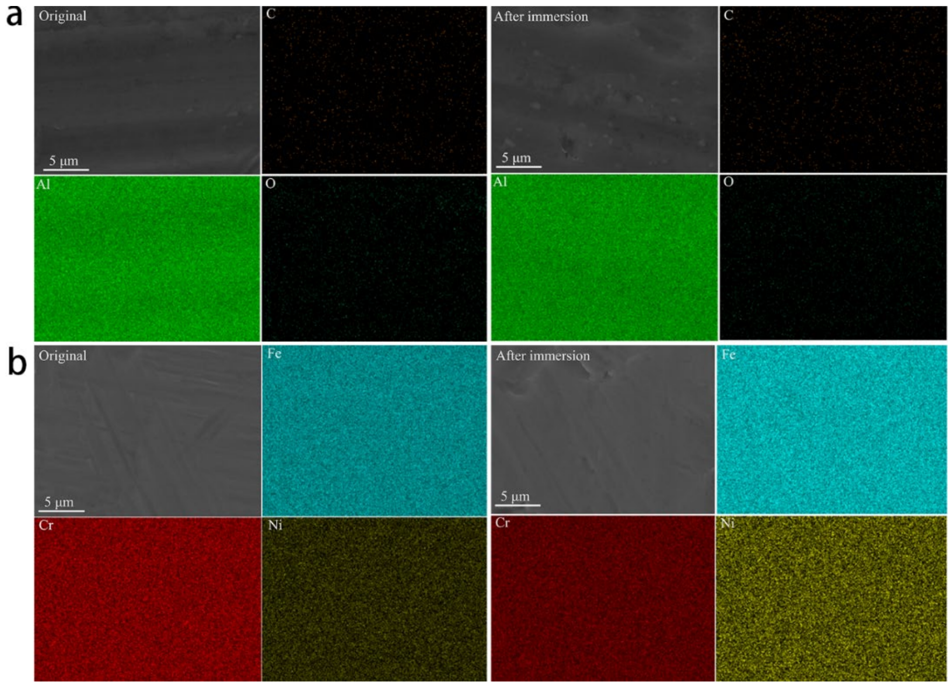

Fig. S6 EDS Mapping surface scanning results of the (**a**) aluminum foil and (**b**) stainless steel before and after immersion in AMHEE

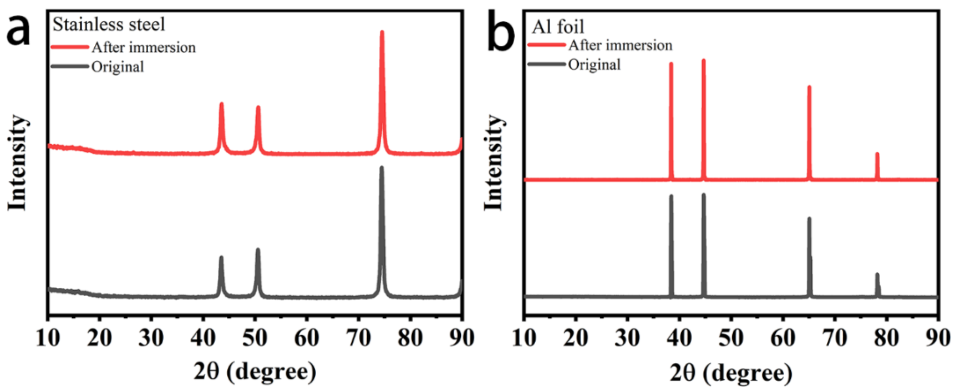

Fig. S7 XRD of (**a**) the stainless steel and (**b**) aluminum foil before and after immersion in AMHEE

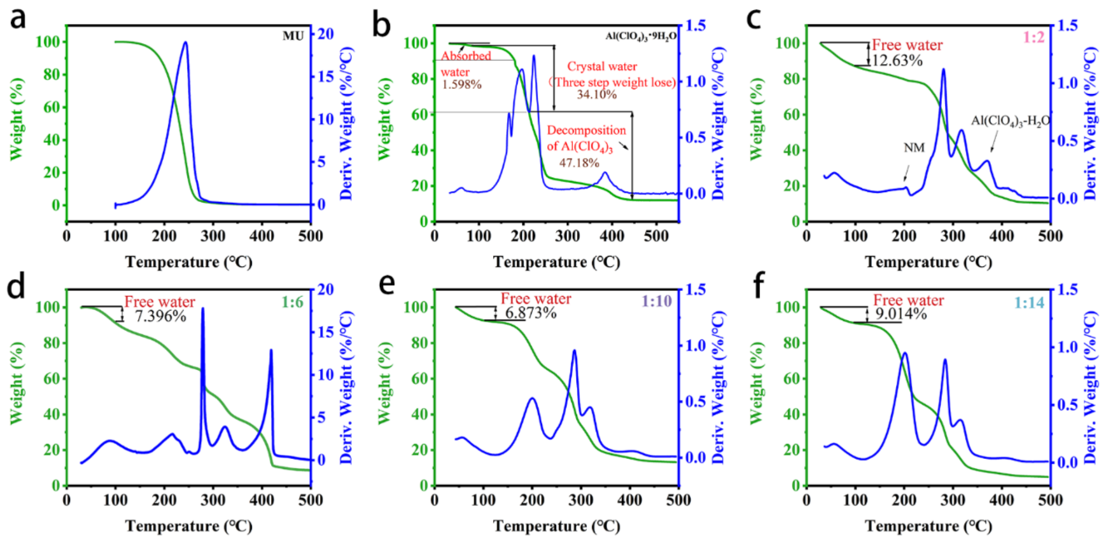

Fig. S8 Thermogravimetric analysis of (**a**) MU, (**b**) $Al(ClO_4)_3\cdot9H_2O$, (**c**) AMHEE-1:2, (**d**) AMHEE-1:6, (**e**) AMHEE-1:10 and (**f**) AMHEE-1:14

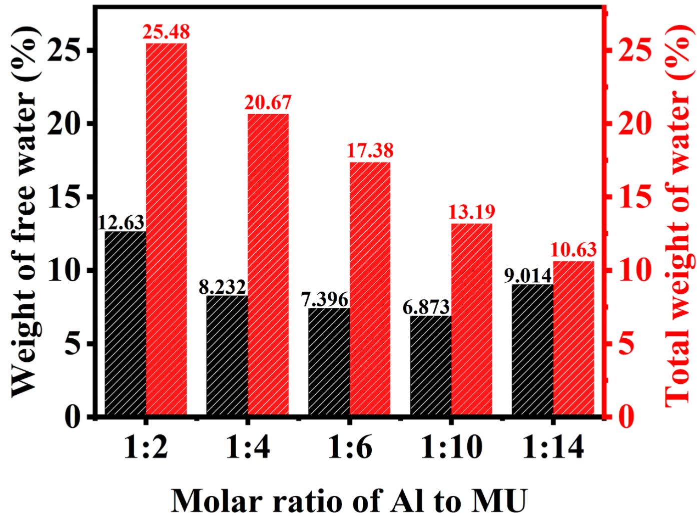

Fig. S9 Gravimetric analysis of water in AMHEE

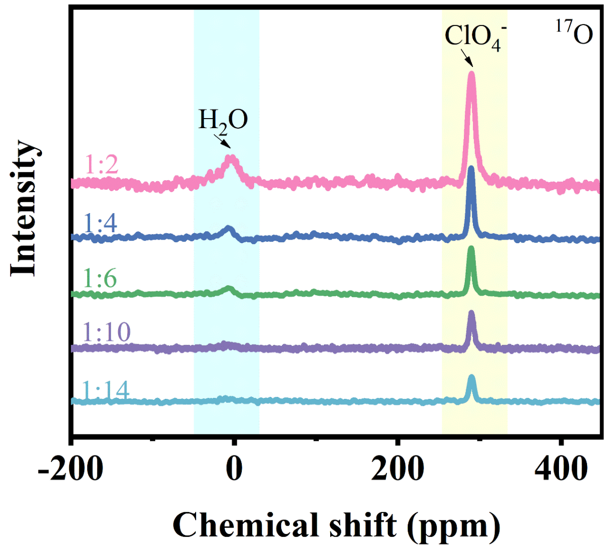

Fig. S10 ^{17}O NMR spectra of AMHEEs with different molar ratios (1:2, 1:4, 1:6, 1:10 and 1:14)

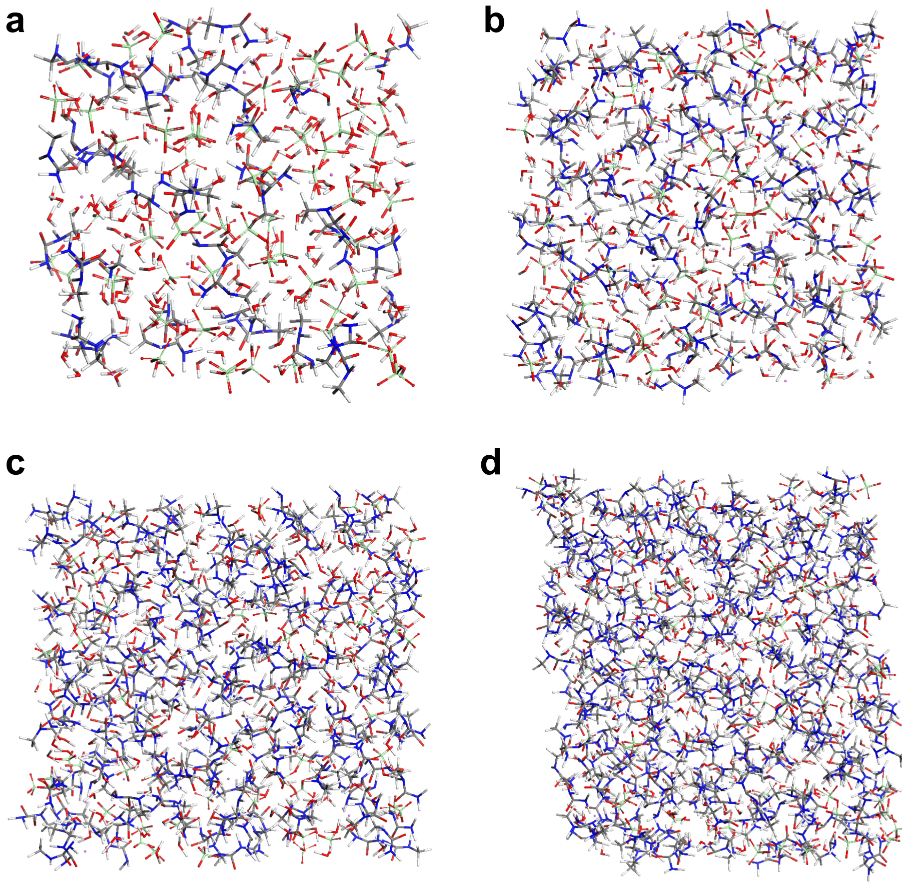

Fig. S11 Solvation structures of $Al(ClO_4)_3 \cdot 9H_2O$/MU mixtures with molar ratios of (**a**) 1:2, (**b**) 1:6, (**c**) 1:10, and (**d**) 1:14

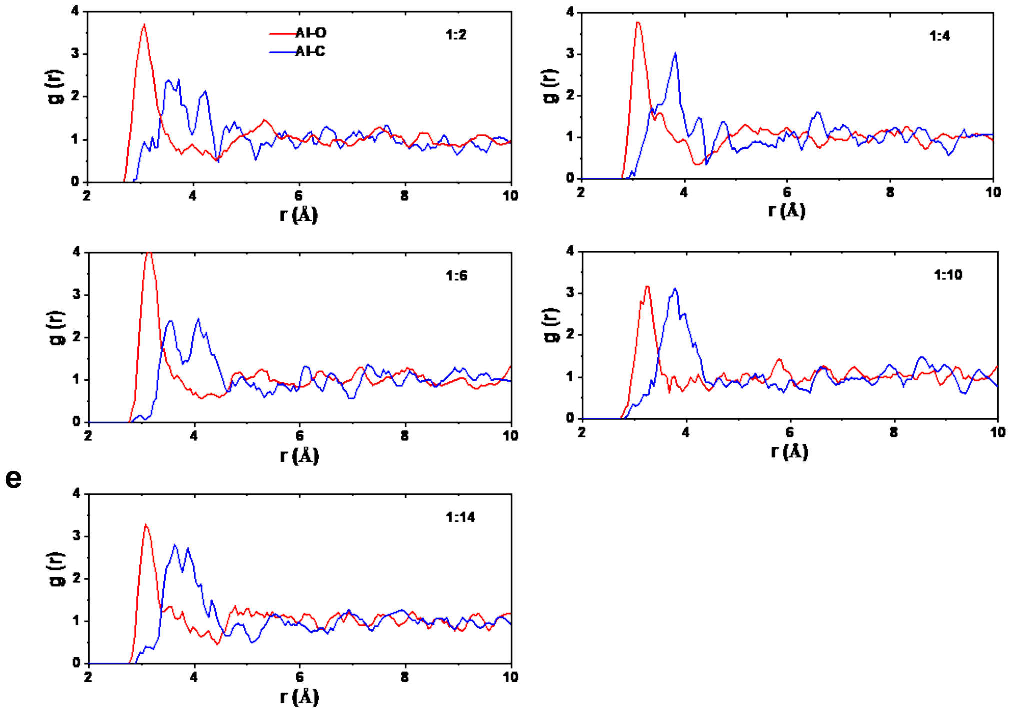

Fig. S12 RDFs of the AMHEEs with ratios of (**a**) 1:2, (**b**) 1:4, (**c**) 1:6, (**d**) 1:10, and (**e**) 1:14 from MD simulations

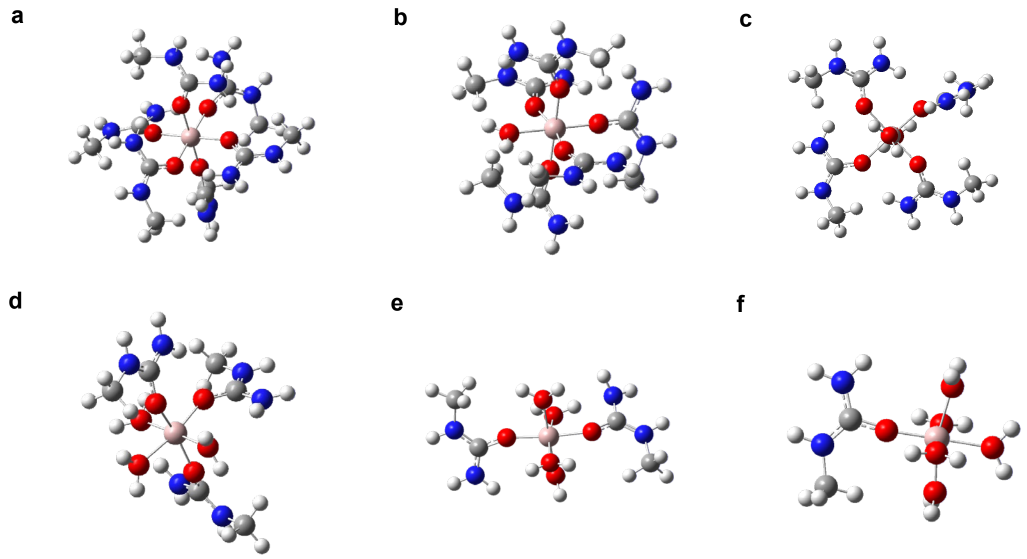

Fig. S13 Optimized structures of Al-complex with coordination number of six through DFT calculations

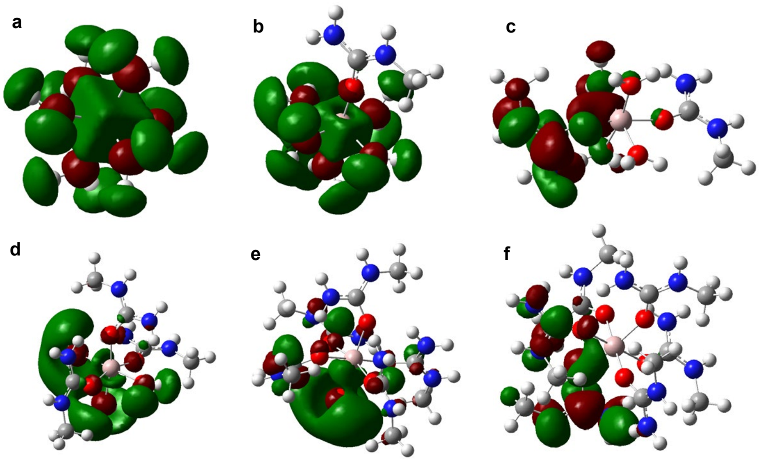

Fig. S14 LUMO partial charge densities of Al-complexes

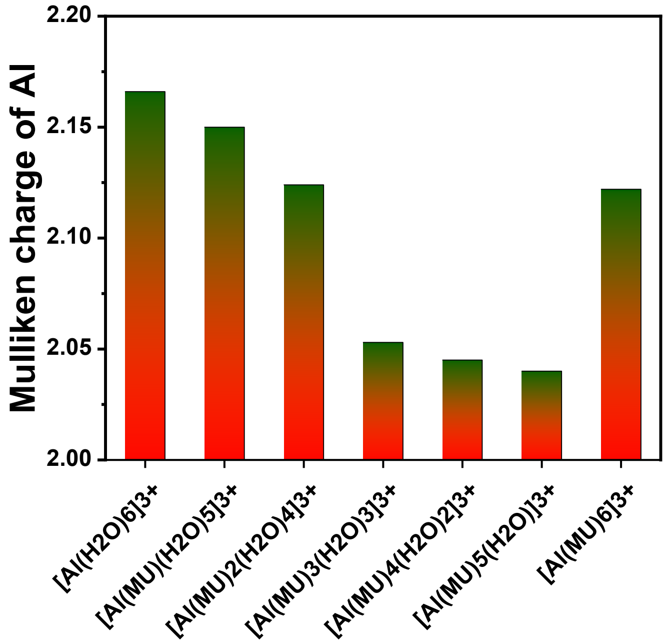

Fig. S15 Mullikan charge of Al in different Al-complexes

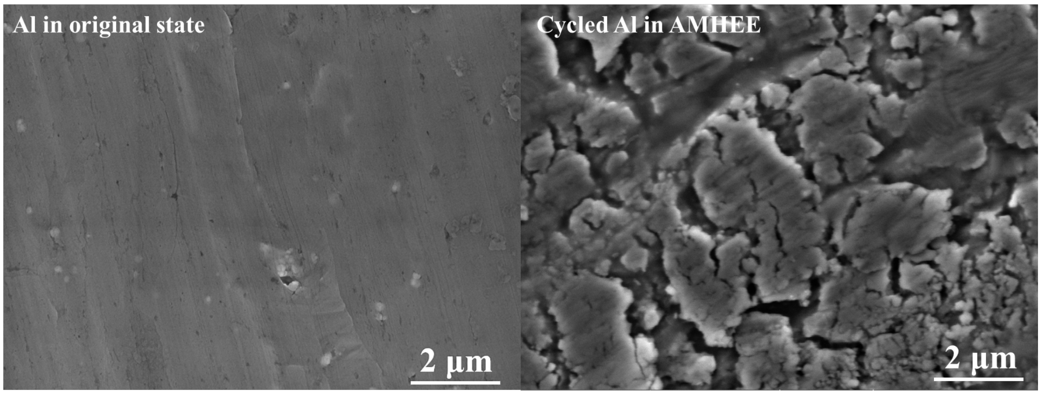

Fig. S16 SEM images of Al in original state (left) and after cycling (right)

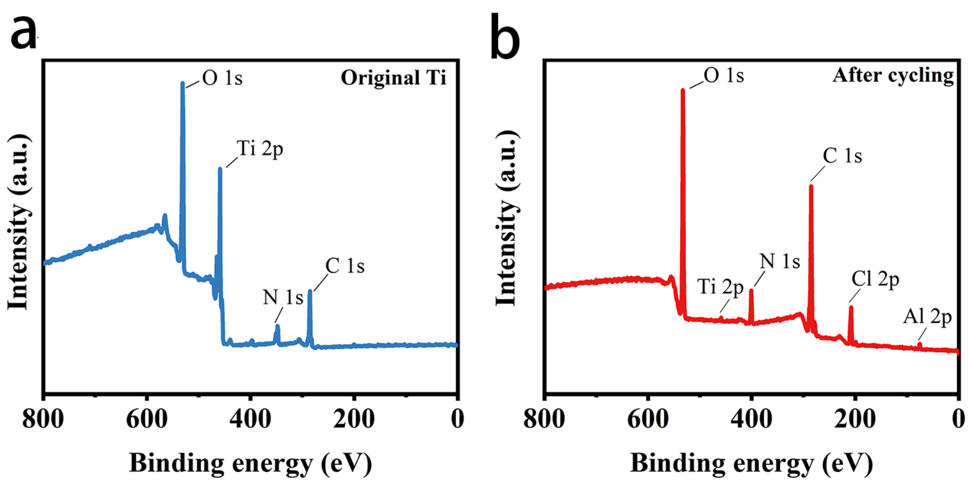

Fig. S17 XPS full spectra of (**a**) original Ti and (**b**) cycled Ti in AMHEE

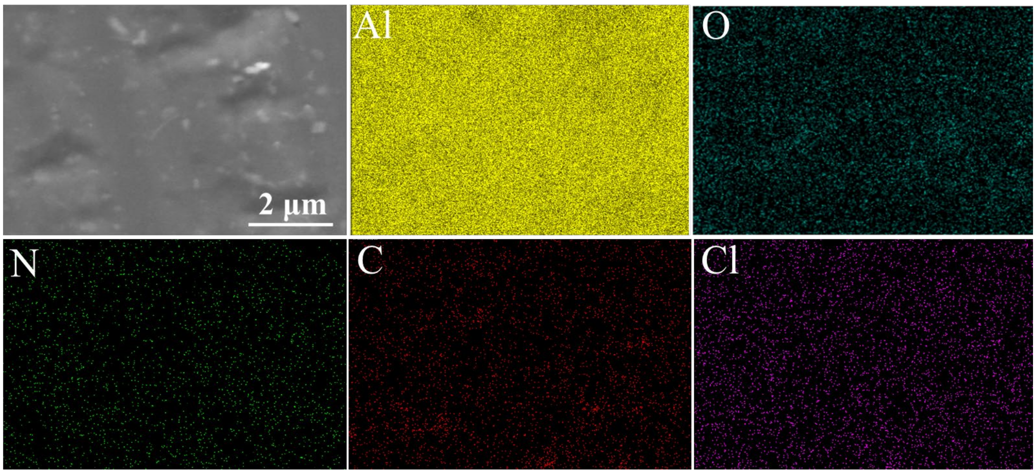

Fig. S18 Elemental mappings of the Al surface after cycling

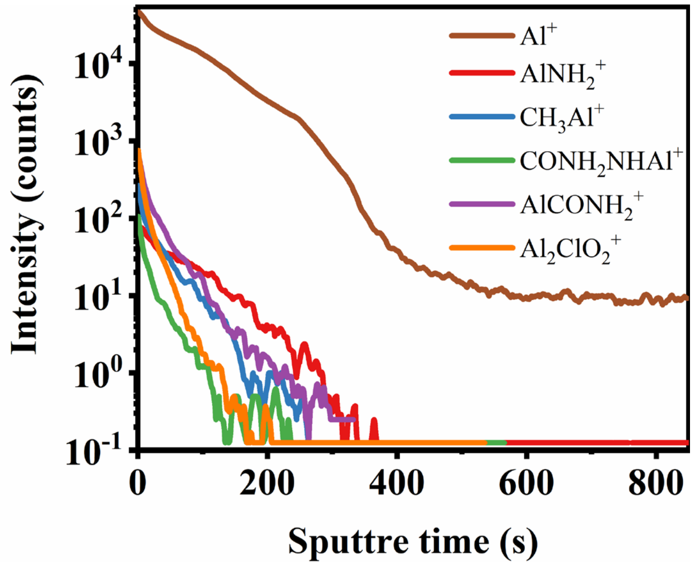

Fig. S19 TOF-SIMS depth profiles of various species of interest acquired from the Al surface

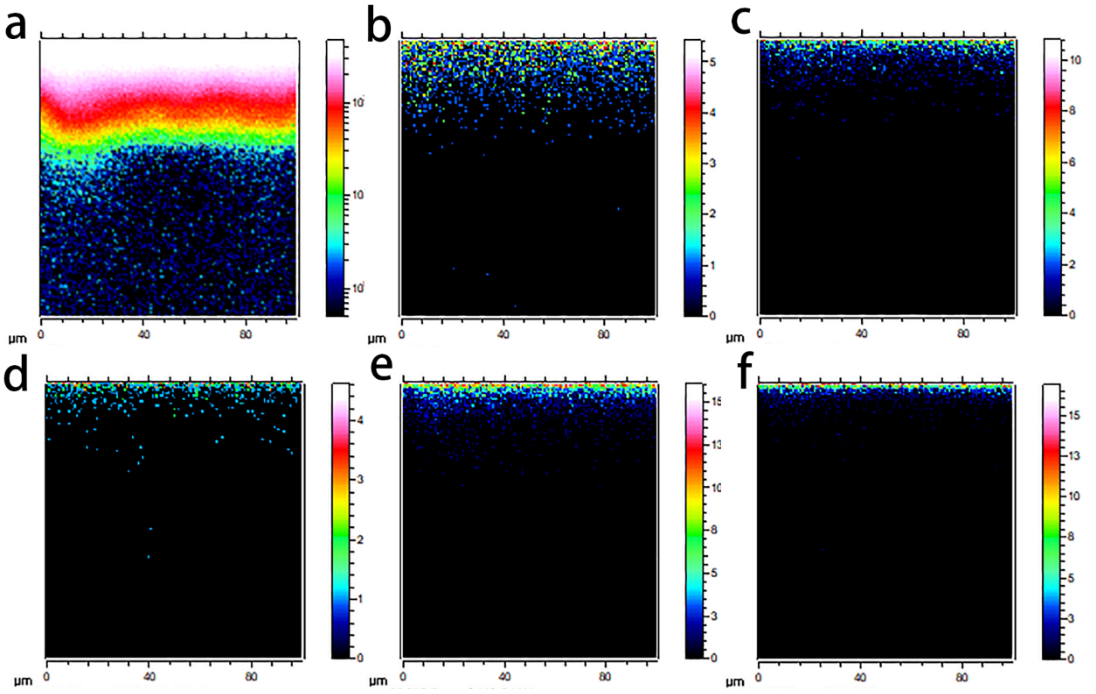

Fig. S20 3D cross-section images of various species of interest acquired from the Al anode. (**a**) Al^{+}, (**b**) $AlNH_2^{+}$, (**c**) CH_3Al^{+}, (**d**) $CONH_2NHAl^{+}$, (**e**) $AlCONH_2^{+}$ and (**f**) $Al_2ClO_2^{+}$

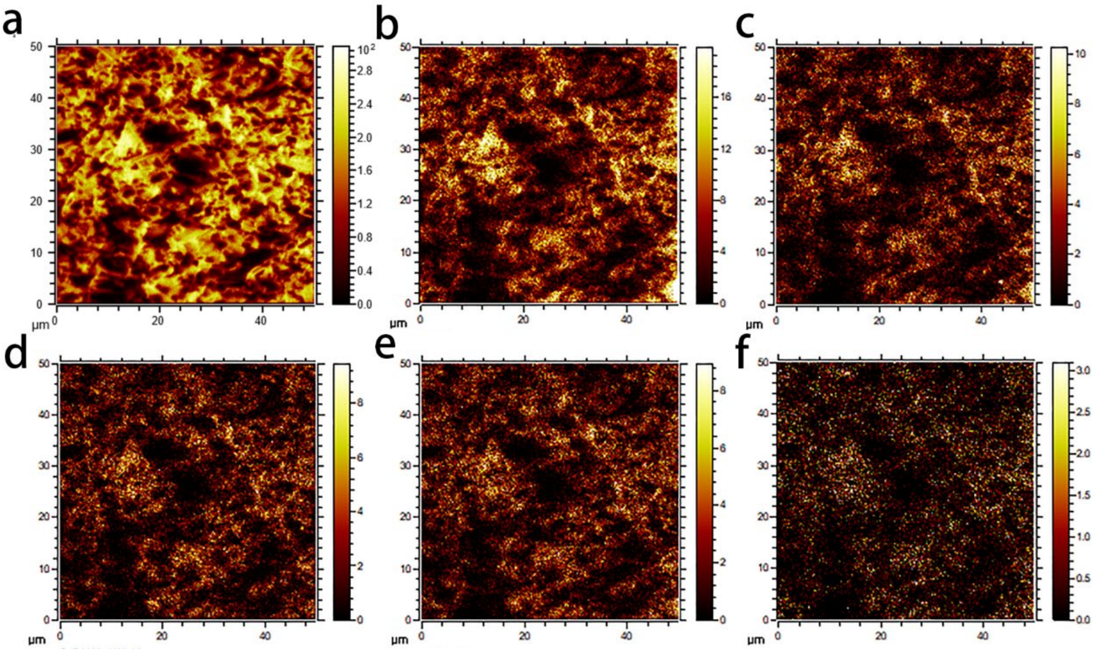

Fig. S21 3D images of component distribution acquired from the Al surface. (**a**) Al^{+}, (**b**) $AlNH_2^{+}$, (**c**) CH_3Al^{+}, (**d**) $CONH_2NHAl^{+}$, (**e**) $AlCONH_2^{+}$ and (**f**) $Al_2ClO_2^{+}$

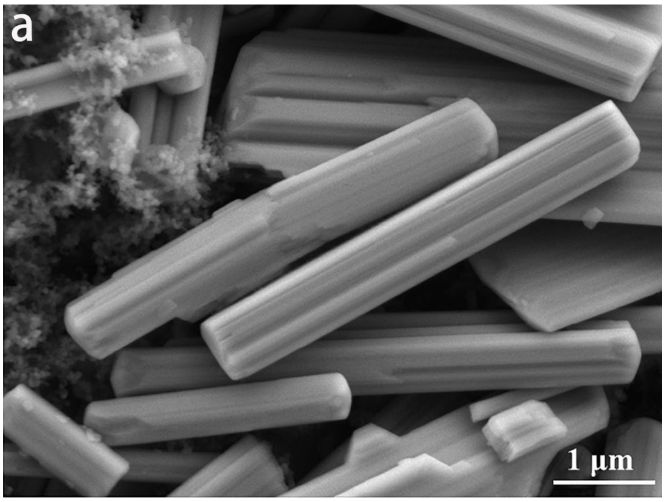

Fig. S22 SEM images of V_2O_5 rods synthesized by hydrothermal method

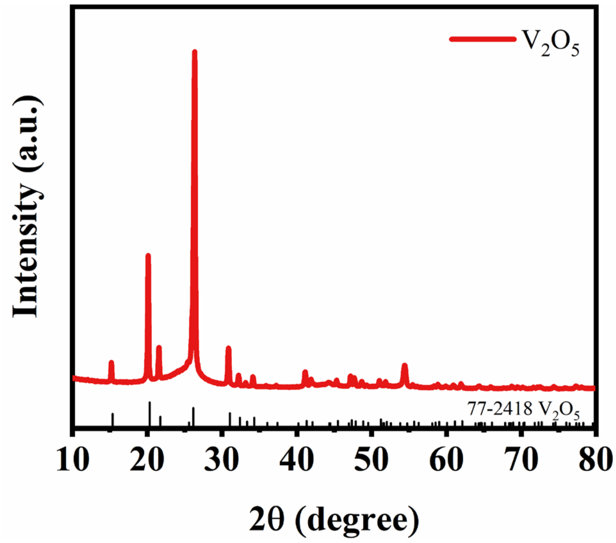

Fig. S23 XRD pattern of the V_2O_5 electrode

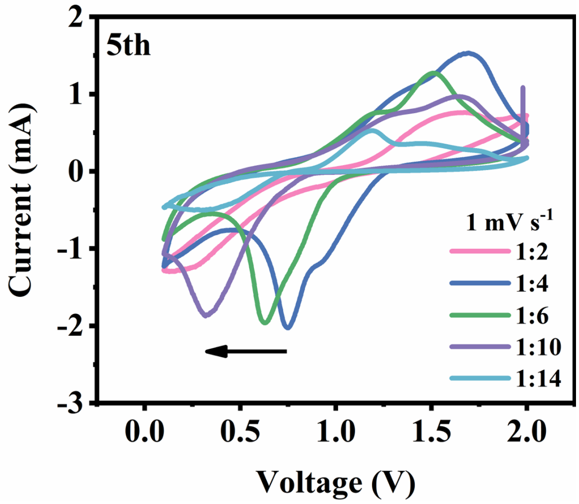

Fig. S24 CV curves of the Al/AMHEE/V_2O_5 full cell with different AMHEEs electrolytes at a scan rate of 1 mV s^{-1}

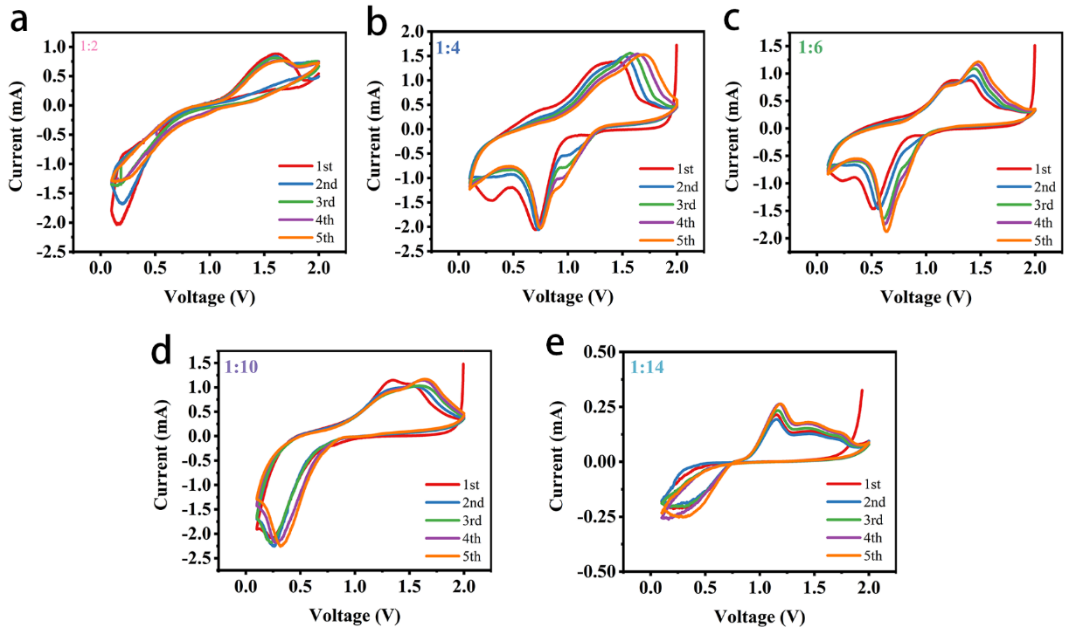

Fig. S25 CV curves of the AIBs. (**a**) AMHEE-1:2, (**b**) AMHEE-1:4, (**c**) AMHEE-1:6, (**d**) AMHEE-1:10 and (**e**) AMHEE-1:14 as electrolytes at a scan rate of 1mV s^{-1}

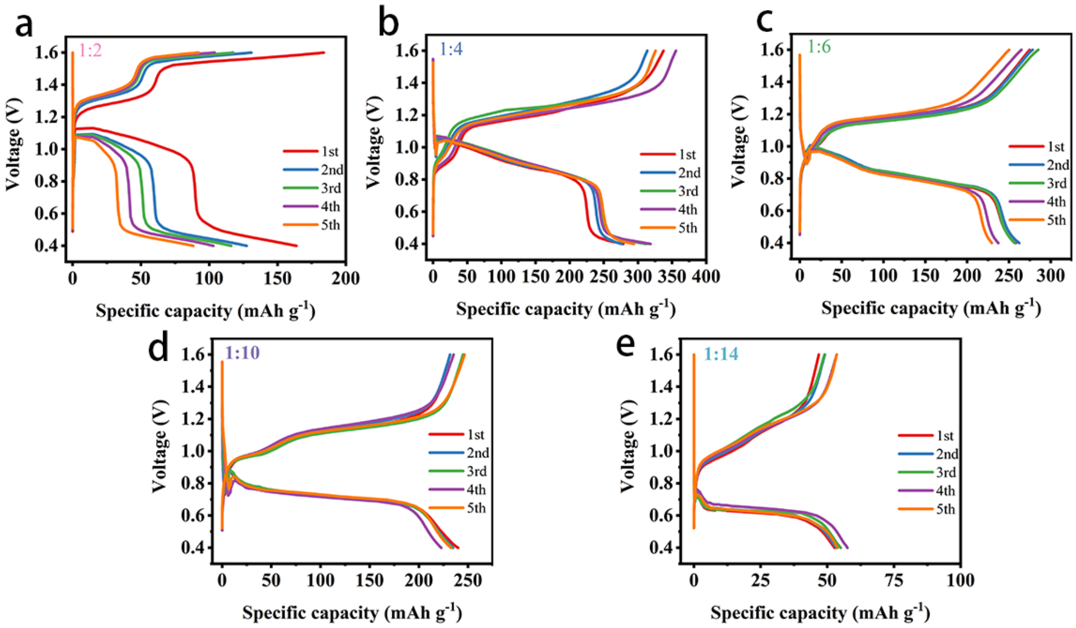

Fig. S26 The discharge/charge curves of AIBs. (**a**) AMHEE-1:2, (**b**) AMHEE-1:4, (**c**) AMHEE-1:6, (**d**) AMHEE-1:10 and (**e**) AMHEE-1:14 as electrolytes at a current density of 0.1 A g^{-1}

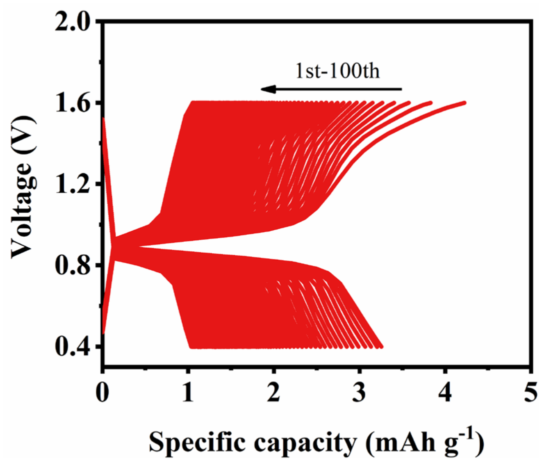

Fig. S27 Charge/discharge curves of the AIB when the carbon paper current collector was used as the positive electrode

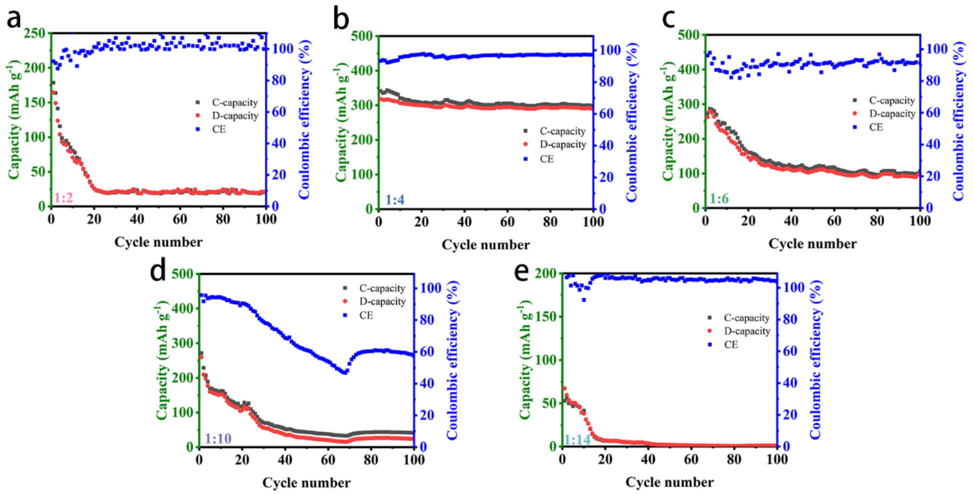

Fig. S28 Cycling performance of AIBs. (**a**) AMHEE-1:2, (**b**) AMHEE-1:4, (**c**) AMHEE-1:6, (**d**) AMHEE-1:10 and (**e**) AMHEE-1:14 as electrolytes at a current density of 0.1 A g^{-1}

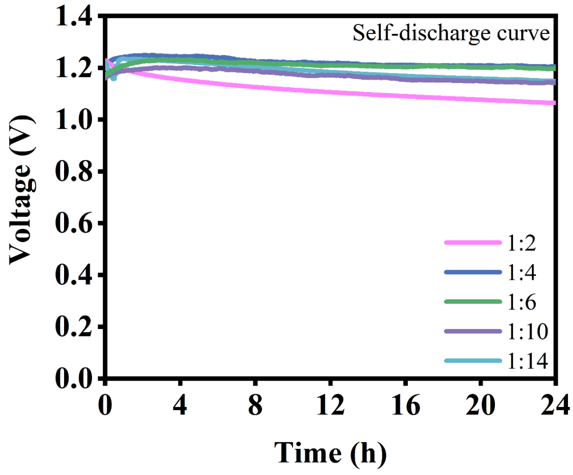

Fig. S29 The self-discharge curves of the AIBs with V_2O_5 in AMHEEs with different molar ratios

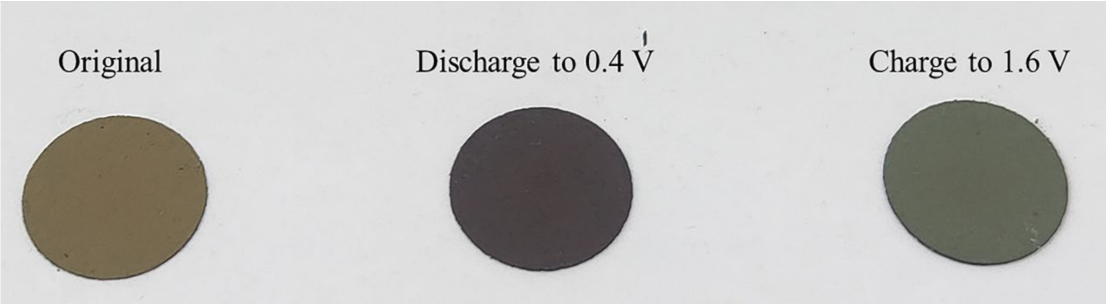

Fig. S30 Photos of V_2O_5 electrodes at different discharge/charge states

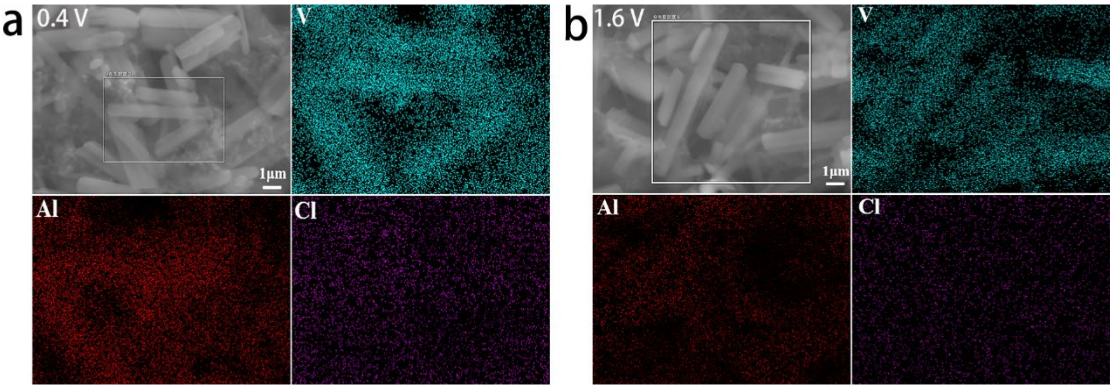

Fig. S31 SEM and EDS mapping images of V_2O_5 electrodes at different voltage states (**a**) 0.4 V and (**b**) 1.6 V

Table S1 Calculated densities and porosities of Al-complex

	1:2	1:4	1:6	1:10	1:14
Density (g/cm^3)	1.675	1.366	1.307	1.232	1.206
Porosity (%)	70.30	78.71	81.54	86.10	86.57

Table S2 Calculated solvation energies of Al^{3+}/MU/H_2O complexes

Complex	E_s (kcal/mol)	E_s (eV)
$[Al(H_2O)_6]^{3+}$	783.1745453	33.96166
$[Al(H_2O)_5]^{3+}$	716.2532288	31.05968
$[Al(H_2O)_4]^{3+}$	644.1607891	27.93345
$[Al(H_2O)_3]^{3+}$	532.7758318	23.10335
$[Al(H_2O)_2]^{3+}$	393.5769369	17.06711
$[Al(H_2O)]^{3+}$	213.0549922	9.238939
$[Al(MU)(H_2O)_5]^{3+}$	832.0049571	36.0791482
$[Al(MU)(H_2O)_4]^{3+}$	781.4009559	33.88475111
$[Al(MU)(H_2O)_3]^{3+}$	723.4292357	31.37085949
$[Al(MU)(H_2O)_2]^{3+}$	632.0501736	27.40828848
$[Al(MU)(H_2O)]^{3+}$	520.4050646	22.56689854
$[Al(MU)]^{3+}$	411.2591741	17.83388496
$[Al(MU)_2(H_2O)_4]^{3+}$	872.8968466	37.85238828

$[Al(MU)_2(H_2O)_3]^{3+}$	835.8180521	36.24449964
$[Al(MU)_2(H_2O)_2]^{3+}$	787.0257737	34.12866628
$[Al(MU)_2(H_2O)]^{3+}$	710.7522495	30.82113336
$[Al(MU)_2]^{3+}$	615.8302386	26.70492556
$[Al(MU)_3(H_2O)_3]^{3+}$	906.569208	39.31256001
$[Al(MU)_3(H_2O)_2]^{3+}$	870.0181489	37.72755614
$[Al(MU)_3(H_2O)]^{3+}$	833.4509064	36.14185049
$[Al(MU)_3]^{3+}$	771.9584156	33.47528382
$[Al(MU)_4(H_2O)_2]^{3+}$	930.5113191	40.35078817
$[Al(MU)_4(H_2O)]^{3+}$	897.7764089	38.93126817
$[Al(MU)_4]^{3+}$	873.0344888	37.85835702
$[Al(MU)_5(H_2O)]^{3+}$	935.5287603	40.56836501
$[Al(MU)_5]^{3+}$	922.9566591	40.02318712
$[Al(MU)_6]^{3+}$	953.6957133	41.35615862

Table S3 Electrochemical performance of AIBs with V_2O_5 cathode

Electrolyte	Current density (mA g^{-1})	1st capacity (mAh g^{-1})	Discharge voltage	Cycle number	Reference
$AlCl_3$/EMImCl IL	125	305	~0.6 V	20	[1]
$Al(OTF)_3$ aqueous electrolyte	40	200	~0.8 V	50	[2]
$Al(ClO_4)_3$•$9H_2O$/MU HEE	100	320	~ 0.9 V	100	This work

Supplementary References

[S1]N. Jayaprakash, S. K. Das, L. A. Archer. The rechargeable aluminum-ion battery. Chem. Commun. **47**, 12610-12612 (2011). https://doi.org/10.1039/C1CC15779E

[S2]Q. Zhao, L. Liu, J. Yin, J. Zheng, D. Zhang, J. Chen, L. A. Archer. Proton intercalation/de-intercalation dynamics in vanadium oxides for aqueous aluminum electrochemical cells. Angew. Chem., Int. Ed. **59**, 3048-3052 (2020). https://doi.org/10.1002/anie.201912634
